# Neuroprotective effects of *Withania somnifera* in the SH-SY5Y Parkinson cell model

**DOI:** 10.1016/j.heliyon.2021.e08172

**Published:** 2021-10-13

**Authors:** Jeerang Wongtrakul, Thananya Thongtan, Benjawan Kumrapich, Chonticha Saisawang, Albert J. Ketterman

**Affiliations:** a Research Institute for Health Sciences, Chiang Mai University, 110 Intavaroros Road, Sriphum, Muang District, Chiang Mai, 50200, Thailand; b Department of Biochemistry, Faculty of Medicine, Chulalongkorn University, 1873 Rama 4 Road, Pathumwan, Bangkok, 10330, Thailand; c Institute of Molecular Biosciences, Mahidol University, 25/25 Putthamonthol Road 4, Salaya, Nakhon Pathom, 73170, Thailand

**Keywords:** Ashwagandha, SH-SY5Y, 6-Hydroxydopamine, Parkinson's disease, Neuroprotection, Peroxidase activity, Thioltransferase activity, Peroxiredoxin, Vimentin, VGF, Protein glutathionylation

## Abstract

Parkinson's disease is the most frequent neurodegenerative motor disorder. The clinical syndrome and pathology involve motor disturbance and the degeneration of dopaminergic neurons in the substantia nigra. Root extracts of *Withania. somnifera*, commonly called Ashwagandha, contain several major chemical constituents known as withanolides. Studies have shown that *W. somnifera* extracts exhibit numerous therapeutic effects including inflammation and oxidative stress reduction, memory and cognitive function improvement. This study aimed to evaluate the protective effects of KSM-66, *W. somnifera* root extract, on 6-hydroxydopamine (6-OHDA)-induced toxicity in the human neuroblastoma SH-SY5Y cell line, as well as the associated oxidative response protein expression and redox regulation activity focused on S-glutathionylation. SH-SY5Y cells were treated with 6-OHDA preceded or followed by treatment with the KSM-66 extract. Using KSM-66 concentrations ranging from 0.25 to 1 mg/ml before and after treatment of the cells with 6-OHDA has resulted in an increased viability of SH-SY5Y cells. Interestingly, the extract significantly increased glutathione peroxidase activity and thioltransferase activity upon pre- or post- 6-OHDA treatment. KSM-66 also modulated oxidative response proteins: peroxiredoxin-I, VGF and vimentin proteins upon 6-OHDA pre/post treatments. In addition, the extract controlled redox regulation via S-glutathionylation. Pre-treatment of SH-SY5Y cells with KSM-66 decreased protein-glutathionylation levels in the cells treated with 6-OHDA. The rescue of mitochondria with 0.5 mg/ml KSM-66 extract showed an increase in ATP levels. These findings suggest that *W. somnifera* root extract acts as a neuroprotectant, thereby introducing a potential agent for the treatment or prevention of neurodegenerative diseases.

## 1 Introduction

Parkinson's disease (PD) is the fastest growing neurological disorder in the world. In 2016, there were reported over 6 million cases of PD (Collaborators, 2018). Based on systemic analysis of epidemiological studies between 1990-2016, PD caused approximately 200,000 deaths and 3.2 million Disability-adjusted life years (DALYs). The number of people with PD is expected to double again to over 12 million by 2040. Moreover, increasing longevity, declining smoking rates and increasing industrialization produce conditions that may increase the burden of disease to over 17 million (Dorsey et al., 2018). Unfortunately the estimated 9.4 million PD global population in 2020 is much greater than the previously reported 6 million PD cases in 2016 (Maserejian et al., 2020). This increased rise in PD prevalence estimates suggests the rapidly growing individual and societal burden will become a major problem in the global aging society unless ways to mitigate this disease are discovered.

Currently, there are many reports of herbal medicines that may help treat and/or delay neurodegenerative diseases. One such medicine is Ashwagandha, from the plant *Withania somnifera*, which is used in many indigenous medicine systems, mainly in Ayurveda in India (Rayees and Malik, 2017). *W. somnifera* has been reported to exhibit numerous properties, including antioxidant, memory enhancing, antiparkinsonian, and anti-inflammatory (Kaur et al., 2013; Mishra et al., 2000). *W. somnifera* contains chemical compounds that include alkaloids (isopelletierine, anaferine, cuseohygrine, anahygrine, etc.), steroidal lactones (withanolides, withaferins), saponins, sitoindosides and acylsterylglucosides (Rai et al., 2018). Major bioactive ingredients of *W. somnifera* consists of withanolides, withaferin A, withanolide A, withanolide D and Anaferin (Bhasin et al., 2019). KSM-66, the *W. somnifera* varietal employed in this study had withanolides as the major active ingredients. The use of withanolides for treatment of aspects of Parkinson's disease has been reported previously (Akhoon et al., 2016; Kuboyama et al., 2005; Vegh et al., 2021). Withanolide A was reported to induce regeneration of axons, dendrites, pre- and post-synapses in the neuron of rat Parkinson's disease model (Kuboyama et al., 2005). Withanolide A also showed proteopathies effects by decreasing α-synuclein levels 38% in of NL5901 *Caenorhabditis elegans*, a Parkinson's disease model, suggesting beneficial effects of withanolide A for PD patients (Akhoon et al., 2016). In addition, root extract of *W. somnifera*, containing approximately 12% withanolides, alone or in combination with a water-soluble formulation of coenzyme-Q10 was reported to enhance activation of pro-survival astroglia and inhibited pro-inflammatory microglia. The combined treatment also inhibited oxidative stress, autophagy activation, pro-inflammatory microglia, and activation of pro-survival astroglia in a PD rat model (Vegh et al., 2021). Mechanisms of action of *W. somnifera* have been increasingly studied. *W. somnifera* has been reported to be involved with several anti-oxidant enzymes e.g. catalase, ascorbate peroxidase, peroxidase, glutathione transferase, glutathione peroxidase, monodehydroascorbate reductase *in vivo* and *in vitro* (Ahmed et al., 2018; Mishra et al., 2014; Sabina et al., 2013). *W. somnifera* has also been reported to inhibit mitochondrial damage and increase ATP production (Vidyashankar et al., 2014). The use of *W. somnifera* extracts demonstrated the neuroprotective effects of *W. somnifera* against 6-hydroxydopamine (6-OHDA) induced Parkinsonism in rats (Ahmad et al., 2005). *W. somnifera* extract was found to reverse several parameters tested e.g. lipid peroxidation, reduced glutathione content, glutathione transferase activities, glutathione reductase, glutathione peroxidase, superoxide dismutase, catalase, catecholamine content, dopaminergic D2 receptor binding and tyrosine hydroxylase expression (Dar et al., 2015). 6-OHDA, has been used as the causative agent in PD models for exploring the molecular basis of cytotoxicity and studying neuroinflammation including neuronal death activated by oxidative stress (Hernandez-Baltazar et al., 2017). 6-OHDA induced neuronal damage by accumulating in the cytosol and undergoing rapid auto-oxidation, promoting a high rate of free radical formation consisting mostly of hydrogen peroxide. 6-OHDA also accumulates in mitochondria thereby inhibiting the activity of the electron transport chain by blocking complex I (Blandini et al., 2008; Simola et al., 2007).

Previously several oxidative stress proteins were differentially expressed in SH-SY5Y cells, Parkinson's model cells, treated with 6-OHDA e.g. vimentin, peroxiredoxin (Wongtrakul et al., 2018). In 2D-electrophoresis under reducing conditions, vimentin decreased expression after 6-OHDA treatment whereas peroxiredoxin increased expression. Vimentin was also identified from 2D-proteomics in non-reducing conditions. The protein showed increased glutathionylation upon 6-OHDA treatment (Wongtrakul et al., 2018). Vimentin is classified into the Type III intermediate filament protein. It has been linked to a large variety of pathophysiological conditions e.g. cataracts, Crohn's disease, rheumatoid arthritis, HIV and cancer (Danielsson et al., 2018). Vimentin also constitutes a critical sensor for electrophilic and oxidative stress, which induces extensive reorganization of the vimentin cytoskeletal network (Monico et al., 2019). Vimentin is the biomarker of several brain diseases with increased expression in rat models for Alzheimer's and Parkinson's disease (Levin et al., 2009; Morales et al., 2016). Peroxiredoxins are a very large and highly conserved family of peroxidases that reduce peroxides (Rhee, 2016). Mammalian peroxiredoxin I (Prx I), belongs to the 2-Cys Prx subgroup that have the conserved NH2- and COOH-terminal Cys residues. Prx I forms reaction intermediates that contain an intermolecular disulfide between the NH2- and COOH-terminal Cys residues and the disulfide is subsequently reduced by thioredoxin (Rhee et al., 2001).

Nerve growth factor inducible, VGF, is a PD dominant biomarker which can be detected at the protein and gene level (Cocco et al., 2020; Henderson-Smith et al., 2016). VGF peptides are synthesized from the vgf (non-acronymic) gene that encodes a precursor of approximately 90 kDa whose transcription and secretion is upregulated by neurotrophins (Ferri et al., 2011). VGF peptides are abundant in substantia nigra, cortex, hippocampus and hypothalamus (van den Pol et al., 1994). Several reports suggest that VGF may represent a useful biomarker in PD (Cocco et al., 2010, 2020; Henderson-Smith et al., 2016). 6-OHDA-lesioned rats had decreased VGF in substantia nigra in short-term treatment. The protein completely disappeared at 6 weeks (Cocco et al., 2020). VGF gene down regulation was noted in PD patients and PD patients with dementia (Henderson-Smith et al., 2016). In addition, VGF c-terminal portions were analyzed in the brain of 6-OHDA-lesioned rat models of PD in parallel with blood samples of PD patients. There was a linear correlation between VGF immune reactivity and disease duration, levodopa equivalent dose and olfactory dysfunction (Cocco et al., 2020).

The aim of this study was to evaluate the neuroprotective effects of a *W. somnifera* root extract. This was performed by employing 6-OHDA to induce neurotoxicity in SH-SY5Y cells, a human neuroblastoma cell line. We then measured the associated oxidative response protein expression and redox regulation activity with a focus on protein S-glutathionylation. To elucidate the neuroprotective mechanism we measured percent cell survival, GST antioxidant enzyme activities; thioltransferase and peroxidase activities including GST detoxification activity; CDNB and ATP levels. In addition, the expression of oxidative response proteins; vimentin, peroxiredoxin and VGF proteins, was also determined in the SH-SY5Y cell lysates, as well as the extent of protein-glutathionylation.

## 2 Materials and methods

### 2.1 Ashwagandha water extract preparation

800 mg of KSM-66 Ashwagandha root extract powder (*W. somnifera*), Nootropics Depot USA was suspended in 40 ml of sterile distilled water and stirred at room temperature overnight. The collected extracts were filtered under sterile conditions and stored at -20 °C as aliquots of stock KSM-66 solution.

### 2.2 Cell culture

The study was approved by Institutional Biosafety Committee on Genetic Engineering and Biotechnology, Chiang Mai University (CMUIBC0361003). Human neuroblastoma SH-SY5Y (ATCC® CRL-2266™) cell line was purchased from ATCC and cultured in Dulbecco's Modified Eagle Medium: Nutrient Mixture F-12 (Ham) (1:1) growth medium (Invitrogen, USA) supplemented with 15% fetal bovine serum, 50 U/ml penicillin, 50 μg/ml streptomycin and 1% non-essential amino acid in a 5% CO_2_ incubator at 37 °C. The media was changed after 4 days and cells were ready for testing at day 7. The cells were plated at a density of 8 × 10^5^ cells/ml.

### 2.3 Determination of IC_50_ for KSM-66 and 6-OHDA

In the analysis of neurotoxic properties, KSM-66 stock solution was diluted prior to use. The extract was added to SH-SY5Y cells in a 96- well plate with the final concentrations of 10, 8, 6, 4, 2, 1, 0.75, 0.5 and 0.25 mg/ml. The plate was agitated gently and incubated in a humidified incubator at 37 °C, 5% CO_2_ incubator for 24 h. For the determination of IC_50_ of 6-hydroxydopamine hydrobromide (6-OHDA; > 98% purity, containing ascorbic acid as stabilizer), HPLC grade, (Sigma, St. Louis, MO). SH-SY5Y cells were incubated with different concentrations of 6-OHDA from 3.9 μM to 2,000 μM for 24 h in a 96-well plate. Cell viability was measured using CellTiter 96® AQueous One Solution Cell Proliferation Assay (MTS) (Promega, USA). Dehydrogenase enzymes found in metabolically active cells have the ability to reduce the MTS compound into a formazan product therefore, the amount of colored formazan product was proportional to the number of viable cells. MTS reagent was added into each well and the quantity of formazan present was determined by measuring the absorbance at 490 nm using a spectrophotometer (Spectra MR Microplate spectrophotometer, DYNEXTechnologies, Chantilly, VA).

### 2.4 Analysis of neuroprotective properties

The analysis of neuroprotective effectiveness of KSM-66 in pretreatment and post treatment of SH-SY5Y cells was performed. Stock solution of KSM-66 was diluted in sterile distilled water. 6-OHDA was used as a 10 mM stock solution in 1X PBS (phosphate buffered saline, pH 7.4, Sigma, St. Louis, MO). In the pretreatment assay, various concentrations of KSM-66 were added to 96-well plate and after 24 h incubation at 37 °C, the cells were treated with 6-OHDA, concentration selected from IC_50_ study, to induce oxidative stress and incubated for additional 24 h. In the post-treatment assays, after the cells were plated overnight, KSM-66 was added 24 h after the initial addition of 6-OHDA, and the cells were also incubated at 37 °C for an additional 24 h. Cell viability was measured as absorbance at 490 nm with MTS assay using Cell Titer96®.

### 2.5 Cell treatments and protein preparation for enzyme activity and Western blot

SH-SY5Y cells (1 × 10^6^ cells per treatment group) were cultured in T-25 cm^2^ overnight. Pretreatment and post treatment of KSM-66 were performed using the same protocol as in 96-well plate. The cells were collected at 24 h and washed with PBS. Cell pellets were suspended in lysis buffer, Cell Signaling Technology®, USA, containing 20 mM Tris-HCl, pH 7.5, 150 mM NaCl, 1 mM Na_2_EDTA, 1 mM EGTA, 1% (w/v) Triton X-100, 2.5 mM sodium pyrophosphate, 1 mM β-glycerophosphate, 1 mM Na_3_VO_4_, 1 μg/ml leupeptin with 4 mM NaF, 1.6 μg/ml aprotinin and 0.8 mM PMSF. Cells were incubated for 45 min on ice, disrupted by sonication (4 times for 5 s), and centrifuged for 15 min at 10,000xg (4 °C). Protein concentrations in the supernatant were determined with the Bradford method (Bio-Rad).

### 2.6 Determination of enzyme activities

Glutathione peroxidase activity with cumene hydroperoxide (Sigma-Aldrich, St. Louis, MO) was determined as previously described (Hiratsuka et al., 1997). The co-substrate solution consisted of 50 mM Tris/5mM EDTA pH 7.6, 0.2 mM NADPH (Sigma-Aldrich, St. Louis, MO), 4 mM GSH and 0.13 unit glutathione reductase (GR) (Sigma-Aldrich, St. Louis, MO). After crude extract and co-substrate solution were added to a 96-well plate, the reaction was initiated by the addition of 1.5 mM cumene hydroperoxide. The oxidation of NADPH to NADP^+^ resulted in decreased absorption at 340 nm and was monitored for 3 min using a multimode reader, Clariostar, BMG LABTECH.

Thioltransferase activity with 2-hydroxyethyl disulfide (HED) (Sigma-Aldrich, St. Louis, MO) was determined as previously described (Collinson and Grant, 2003). The activity was measured at 340 nm by the use of GR as a coupling enzyme. The assay mixture contained 1 mM GSH, 6 μg/ml glutathione reductase, 0.7 mM HED, 0.4 mM NADPH and 0.1 M Tris pH 7.5. After crude extract and substrate mixture were added, the decrease in absorbance at 340 nm was recorded for 3 min using a multimode reader.

GST specific activity was determined as previously described (Habig et al., 1974). The substrate mixture contained 0.1 M sodium phosphate buffer pH 6.5, 10 mM GSH (Sigma-Aldrich, St. Louis, MO) and 1 mM 1-chloro-2,4-dinitrobenzene (CDNB) (Sigma-Aldrich, St. Louis, MO). The reaction conducted at 25 °C in a 96-well plate, was initiated by the addition of crude extract and the prepared substrate. The change in absorbance at 340 nm for 1 min was monitored in 96-well plates in a multimode reader.

### 2.7 Western blotting

Cell lysates for Western-blotting were prepared exactly as described above. The lysate protein was made into a concentrate using a Viva-spin 2 ultrafiltration column with a 10 kDa molecular weight cut-off (GE Healthcare, Buckinghamshire, UK) in accordance with the manufacturer's recommendations. The protein concentration was determined as before. A quantity in the range of 10–20 μg of concentrated lysate was resolved by SDS-PAGE and transferred to PVDF membrane. Western blot analysis for detection of peroxiredoxin I, vimentin and VGF proteins was carried out using the following antibodies: anti-peroxiredoxin 1 rabbit polyclonal antibody (Thermo Fisher, USA), anti-vimentin mouse monoclonal antibody (Thermo Fisher, USA) and anti-VGF rabbit polyclonal antibody (Thermo Fisher, USA). The secondary antibodies used were goat anti-rabbit IgG-HRP (Santa Cruz Biotechnology, CA) and anti-mouse IgG (Sigma, USA). ECL detection was performed according to the manufacturer's protocol (Merck KGaA, Darmstadt, Germany). ImageJ software was employed to determine the optical density values of bands for relative comparisons. The experiment was performed in duplicate.

### 2.8 Protein glutathionylation

SH-SY5Y cells were pretreated with or without 0.5 mg/ml KSM-66 for 24 h and then incubated with 100 μM 6-OHDA for 15 min. Cells were harvested by using lysis buffer, Cell Signaling Technology®, USA. Equal amounts of protein (40 μg) in each group were loaded onto a sodium dodecyl sulfate polyacrylamide gel electrophoresis (SDS-PAGE) under non-reducing (DTT was omitted) conditions, immunoblotted as described above and probed with anti-glutathione mouse monoclonal antibody (Abcam, UK). The experiments were performed in three independent experiments with triplicates.

### 2.9 Quantification of ATP content

SH-SY5Y cells were pretreated with or without 0.5 mg/ml KSM-66 for 24 h and then incubated with 100 μM 6-OHDA for 2 h. For post-treatment, SH-SY5Y cells were incubated with 100 μM 6-OHDA for 2 h then incubated with 0.5 mg/ml KSM-66 for 24 h. Cells were collected and washed with PBS. ATP content was measured by a commercial ATP Assay Kit (Colorimetric/Fluorometric) (Abcam, UK) according to the manufacturer's instruction. Briefly, cells (1 × 10^6^) were lysed in 100 μl of ATP assay buffer, homogenized, and centrifuged (13,000 ×g, 5 min, 4 °C) to pellet insoluble materials. The supernatants were collected, transferred to new tubes and deproteinized using Deproteinizing Sample Preparation Kit – TCA (Abcam, UK). Briefly, 10 μl of cold TCA was added into 100 μl sample. The sample tube was placed on ice for 15 min and centrifuged at 12,000 x g for 5 min. The supernatant was transferred into another tube. Samples were diluted at 1:10 in ATP assay buffer. To obtain fluorescence measurements with a standard ATP, 50 μl of supernatant was mixed with 50 μl of ATP reaction mix solution. The standard curve of ATP was obtained by serial dilution of 0.1 mM ATP solution. The plates were incubated at room temperature for 30 min, while being protected from light and fluorescence in the wells was measured at Ex/Em = 535/587 nm using a multimode reader, Clariostar, BMG LABTECH.

### 2.10 Statistics

The data were expressed as mean ± SEM. All IC_50_, cell viability, enzyme activities, western blot data and ATP content were analyzed using the Graphpad Prism program version 5.0 (GraphPad Software Inc., San Diego, CA). Statistical analysis of significance was undertaken by One-Way ANOVA with Dunnett's Multiple Comparison Test including Bonferroni's multiple comparison test. *P* values less than 0.05 were considered statistically significant.

## 3 Results

### 3.1 Determination of IC_50_ for 6-hydroxydopamine and KSM-66

**Figure 1:**
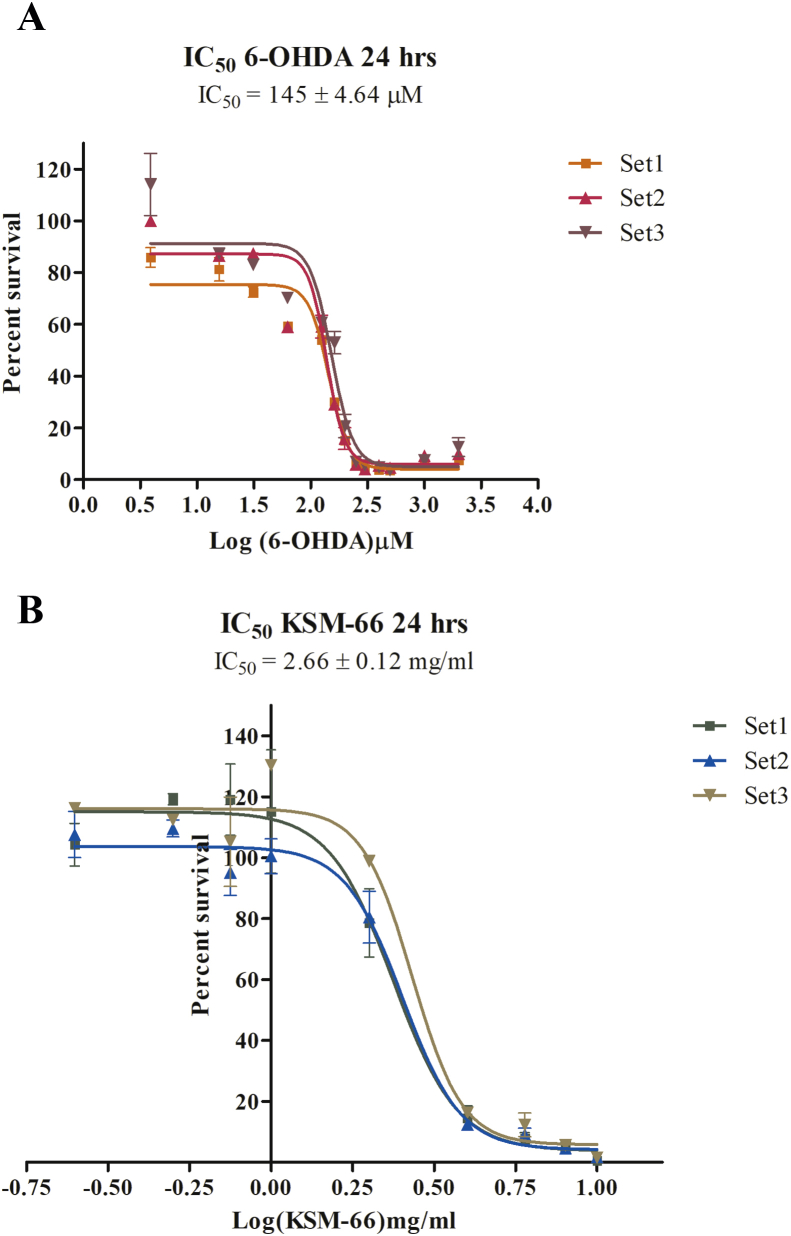
SH-SY5Y cell viability for 6-OHDA and KSM-66 treatment. (A) Concentration of 6-OHDA ranged from 3.9 to 2,000 μM to obtain 50% cell viable (IC_50_) as measured by MTS assay. The IC_50_ value concentration as shown is at 145 μM. (B) Concentration of KSM-66 ranged from 0.25 to 10 mg/ml to obtain 50% cell viable (IC_50_) as measured by MTS assay. The IC_50_ value concentration as shown is at 2.66 mg/ml. The control is measured as 100% viability. The values are presented as mean ± SEM.

**Figure 2:**
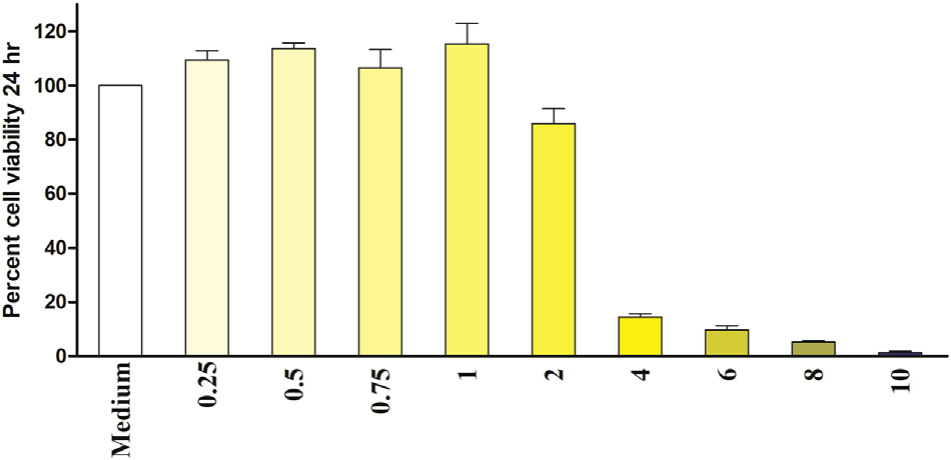
Cytotoxicity test of KSM-66 towards SH-SY5Y cells as measured by MTS assay after 24 h of incubation at 37 °*C incubator*. Toxicity effect can be seen in the concentration range of 4–10 mg/ml. Error bars show SEM of at least three experiments performed in duplicate. Data were analyzed by One-Way ANOVA with Dunnett's multiple comparison test; ∗*p* < 0.05. ∗indicates significant difference.

To determine the appropriate concentrations of dopaminergic neurotoxin 6-OHDA and KSM-66 Ashwagandha root extract, SH-SY5Y cells were cultured overnight and then were exposed to a series of concentrations from 6-OHDA ranging from 3.9 μM to 2,000 μM whereas KSM-66 concentrations range from 0.25 mg/ml to 10 mg/ml. The cells were exposed to 6-OHDA or KSM-66 for 24 h. The mean ± SEM IC_50_ values of 6-OHDA and KSM-66, determined by MTS assay, were 145 ± 4.64 μM and 2.66 ± 0.12 mg/ml respectively (Figure 1). As shown in Figure 2, KSM-66 extract was cytotoxic at concentrations greater than 1 mg/ml. One-way ANOVA with Dunnett's Multiple Comparison Test showed a significant effect of KSM-66 at 4–10 mg/ml concentrations (*p* < 0.05) as compared to control. Therefore, KSM-66 at 1 mg/ml was chosen for further experiments.

### 3.2 Analysis of neuroprotective properties

**Figure 3:**
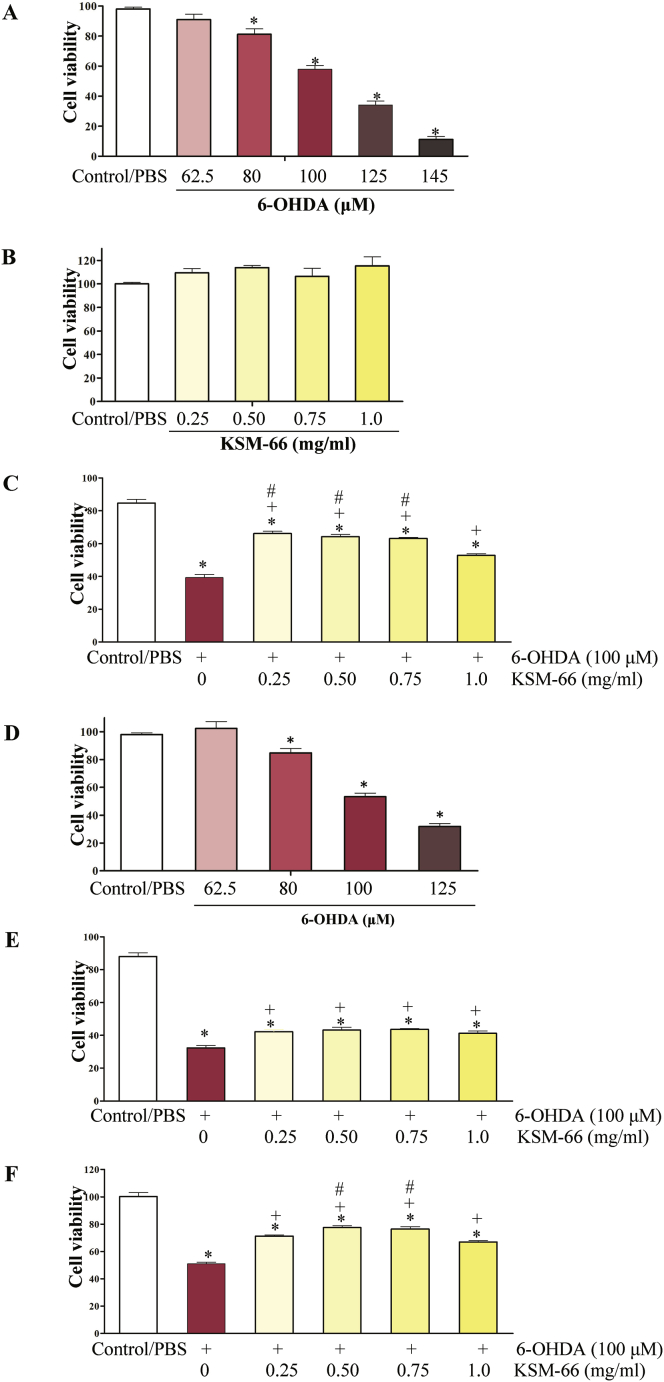
Effects of 6-OHDA and KSM-66 on SH-SY5Y cell viability in pre/post-treatment assays. First we performed post treatment experiments shown in A-C. (A) 6-OHDA could decrease viability. (B) KSM-66 at 0.25, 0.5, 0.75 and 1.0 mg/ml has no toxicity to the cells (C) The effects of KSM-66 on 6-OHDA mediated cellular damage are presented. The KSM-66 extract was added 24 h after exposure to 6-OHDA. . We then measured the effects of 6-OHDA and KSM-66 on SH-SY5Y cell viability in pre-treatment assay as shown in D-F. (D) Increasing 6-OHDA concentration decreased cell viability. (E) The effects of KSM-66 on 6-OHDA mediated cellular damage. The KSM-66 extract was added 30 min before exposure to 6-OHDA. (F) The extract was added 24 h before exposure to 6-OHDA. The values are presented as mean ± SEM (n = 6). Data were analyzed by One-way ANOVA with Bonferroni's Multiple Comparison test; ∗*p* < 0.05 represents significant differences compared to the control, ^+^*p* < 0.05, represents significant differences compared to 6-OHDA exposed cells and ^#^*p* < 0.05 represents significant differences compared to 1 mg/ml KSM-66 exposed cells.

To evaluate the neuroprotective effects of the KSM-66 extract on cell viability upon oxidative stress treatment, the MTS assay was performed. In post-treatment study, 6-OHDA concentrations ranged from 62.5 to 145 μM were tested without KSM post-added for more 24 h. It was found that 6-OHDA decreases cell viability in a dose-dependent manner. In groups receiving 80, 100, 125 and 145 μM of 6-OHDA, the toxicity was statistically significantly different from control SH-SY5Y. Data is expressed in term of mean ± SEM (N = 6), significant changes are given as ∗ (p < 0.05) as compared to control. The concentration at 100 μM of 6-OHDA (relative cell viability, 57.89 ± 2.59%) was used to induce toxicity. Therefore, this concentration was chosen to examine the effects of the extracts (Figure 3A). The concentration of KSM-66 at 0.25, 0.50, 0.75 and 1.0 mg/ml had no effect on cell viability in post-treatment study (Figure 3B). The cells were exposed to 6-OHDA for 24 h of incubation and later exposed to KSM-66 for a further 24 h (Figure 3C). Interestingly, the incubation of the extracts at 0.25–1 mg/ml partially inhibited 6-OHDA mediated toxicity. As shown in Figure 3C, all the concentrations tested had greater significant differences compared to the 6-OHDA only treated sample (*p* < 0.05). KSM-66 concentrations of 0.25–0.75 mg/ml gave greater protective effects compared to the 1 mg/ml treated group (*p* < 0.05).

In the pre-treatment experiment, 6-OHDA concentrations were tested without KSM for 24 h. The concentration at 80, 100 and 125 μM was toxic to the cells showing significantly different cell viability compared to control. The concentration at 100 μM showed 53.22 ± 2.59% cell viability and was chosen for the pre-treatment study (Figure 3D). KSM-66 concentration ranging from 0.25 to 1 mg/ml was added 30 min or 24 h before exposure to 100 μM 6-OHDA (Figure 3E and F). The results showed that all KSM-66 tested concentrations gave significantly greater cell survival compared to the 6-OHDA treated group at both 30 min and 24 h pre-treatment times (*p* < 0.05). In addition, 24h pre-treatment prior to oxidative stress induction gave greater cell survival compared to the 30 min pre-treatment. All KSM-66 dosages at 30 min exposure provided similar extents of protection. However, at 24 h exposure it was found that the 1 mg/ml KSM-66 treated group showed a decreased ability to protect the cells compared to 0.5 and 0.75 mg/ml treatment (*p* < 0.05). The cells showed the most viability at 0.5 mg/ml 24 h pre-treatment with approximately a 30% increase in cell survival compared to 6-OHDA treated group (*p* < 0.05).

### 3.3 Effect of KSM-66 on antioxidant enzyme activity in 6-OHDA induced toxicity

**Figure 4:**
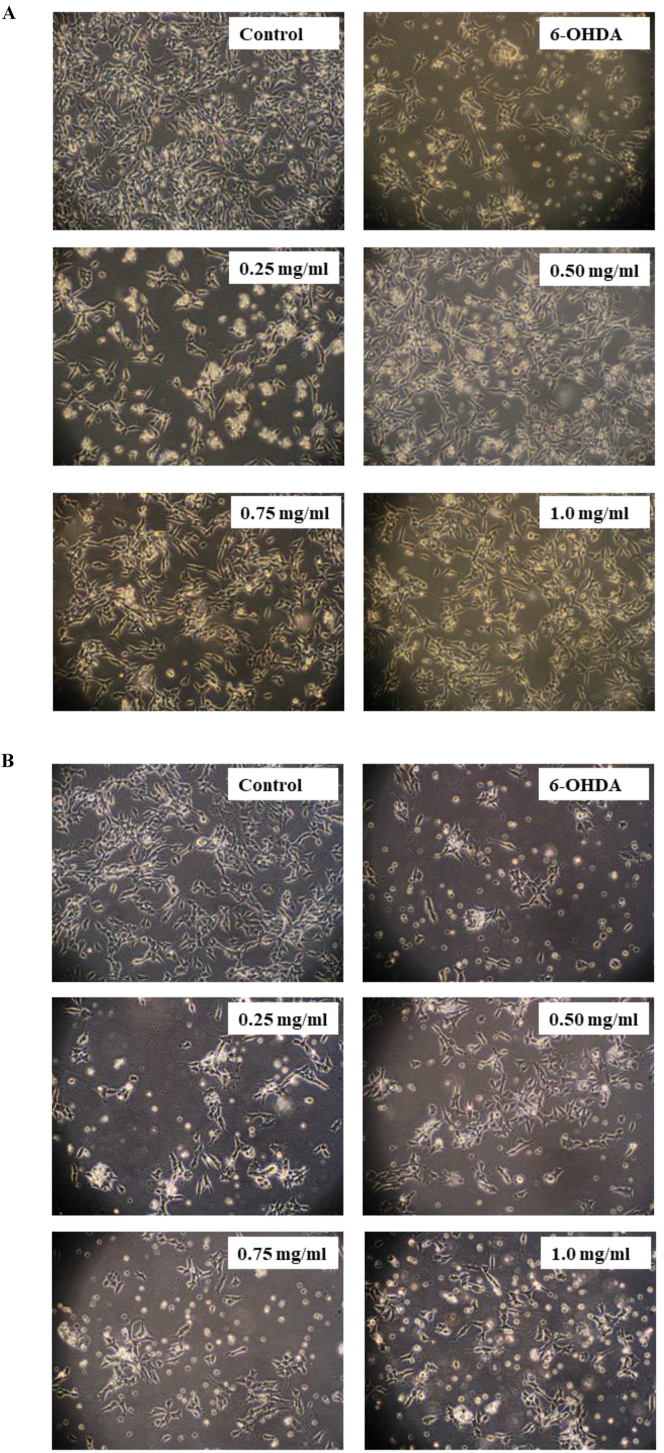
Effects of pre/post-treatment with KSM66 against 6-OHDA-induced oxidative stress in SH-SY5Y cells. (A) Morphology of SH-SY5Y treated with 6-OHDA in the absence or presence of pre-treated KSM-66 (0.25, 0.50, 0.75 and 1.0 mg/ml) for 24 h. (B) Morphology of SH-SY5Y treated with 6-OHDA in the absence or presence of post-treated KSM-66 (0.25, 0.50, 0.75 and 1.0 mg/ml) for 24 h. Representative morphology was determined by Olympus light microscope with magnification x200 microscopy. Normal morphology of SH-SY5Ycells is shown in the control.

**Figure 5:**
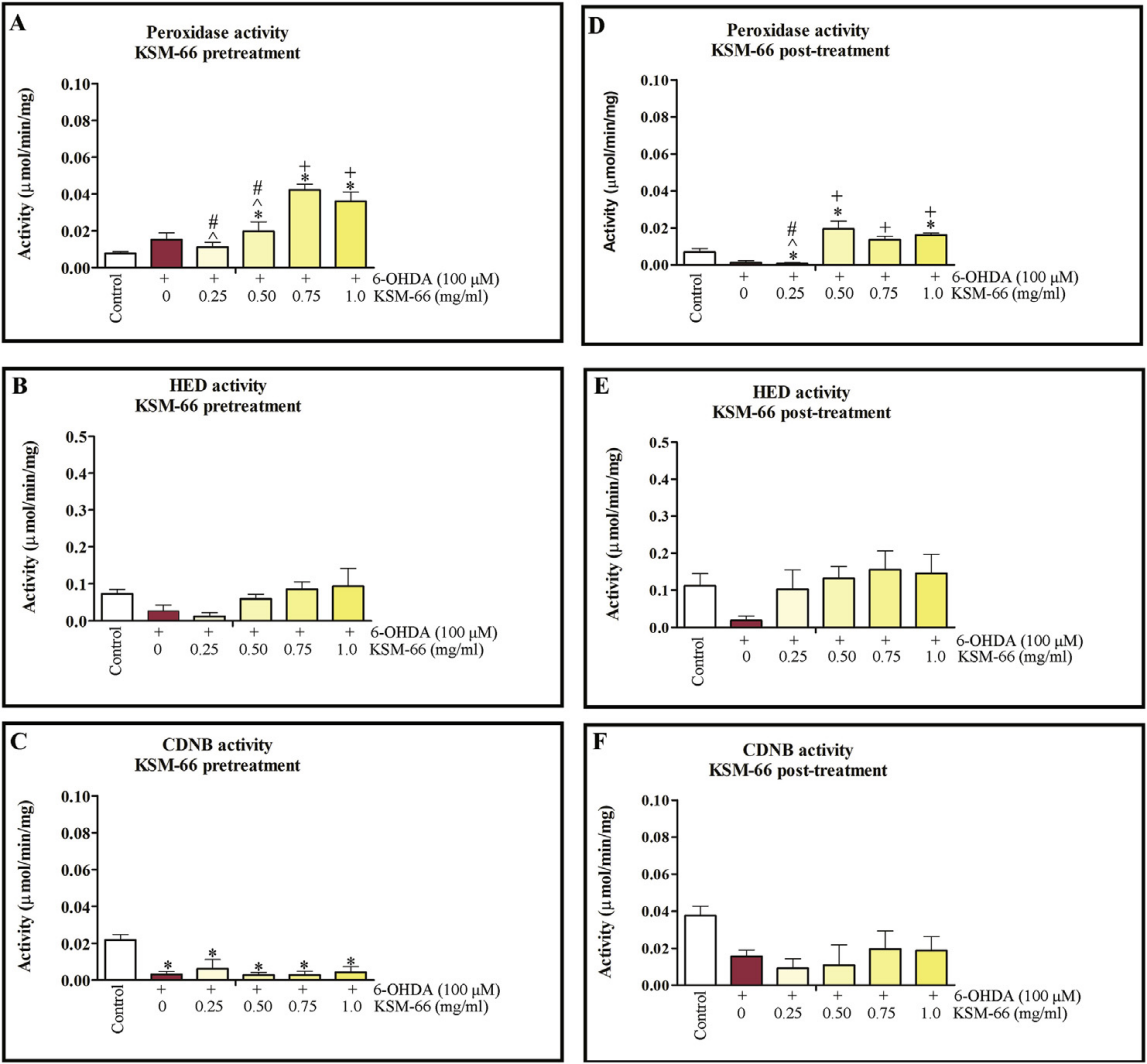
Pre/post-treatment effect of KSM-66 on several enzymes in SH-SY5Y cell lysate. In pre-treatment, the KSM-66 extract was added 24 h prior to 6-OHDA exposure. Pre-treatment effects are shown for (A) glutathione peroxidase (GPx), (B) thiol transferase and (C) CDNB activities. In the post-treatment study, the KSM-66 extract was added 24 h after exposure to 6-OHDA. The post-treatment study show 6-OHDA reduces glutathione peroxidase (GPx) (D), thiol transferase (E) and CDNB (F) activities, whereupon KSM-66 increases their activity. Bar graphs shows mean ± SEM of GPx, thiol transferase and CDNB activities of all groups. The values are presented as mean ± SEM. Data were analyzed by One-way ANOVA with Bonferroni's Multiple Comparison test; ∗*p* < 0.05 represents significant differences compared to the control; ^+^*p* < 0.05 represents significant differences compared to 6-OHDA exposed cells; ^#^*p* < 0.05 represents significant differences compared to 1 mg/ml exposed cells; ^ˆ^*p* < 0.05 represents significant differences compared to 0.75 mg/ml exposed cells.

We next investigated the effect of KSM-66 on the antioxidant defense, thioltransferase activity and detoxification in SH-SY5Y. Neuroprotective properties of KSM-66 in 6-OHDA-induced oxidative stress model of SH-SY5Y cells were investigated in pre-treatment and post-treatment experiments. Morphological changes were also observed in 6-OHDA-treated cells with the 6-OHDA-induced morphological changes restored and prevented by treatment with KSM-66 (Figure 4). Pre-treatment of the cells with KSM-66 resulted in intact cell morphology compared to post-treatment. The effect of KSM-66 pre/post-treatment on the GST activities for cumene hydroperoxide, HED and CDNB substrates were monitored. For the pre-treatment study, 6-OHDA treatment tended to increase glutathione peroxidase, but decrease thioltransferase and CDNB activities (Figure 5A, B and C). Interestingly, peroxidase activity was significantly recovered by 0.75 and 1 mg/ml KSM-66 treatment compared to 6-OHDA exposed cells. 6-OHDA treated cells showed reduced levels of thioltransferase activity compared to control. The treatment groups increased thioltransferase activities to similar levels as the untreated control. CDNB activity of GST in the cells treated with 6-OHDA decreased significantly compared to control. The Ashwagandha extract did not increase CDNB activity in the treatment groups. Post-treatment of the cells with 1 mg/ml KSM significantly increased peroxidase activity compared to 6-OHDA cells (Figure 5D). 6-OHDA treated cells had reduced levels of thioltransferase activity compared to control (Figure 5E). However, treatment with KSM-66 increased the amounts of thiol transferase activities to similar amounts as the untreated control. CDNB activity of GST from cell lysates with KSM-66 post-treatment were similar to 6-OHDA treated cells, that is, showing no rescue effects (Figure 5F).

### 3.4 Effect of KSM-66 on oxidative stress protein expression

To determine the role of KSM-66 in neuroprotection, we studied possible changes in peroxiredoxin I, VGF and vimentin expression by analyzing lysates of 6-OHDA treated SH-SY5Y cells as a model of Parkinson with pre/post treatment with 0.5 mg/ml KSM-66 for 24 h. Western blotting was performed using peroxiredoxin I, VGF and vimentin antibodies that specifically bind to those proteins.

**Figure 6:**
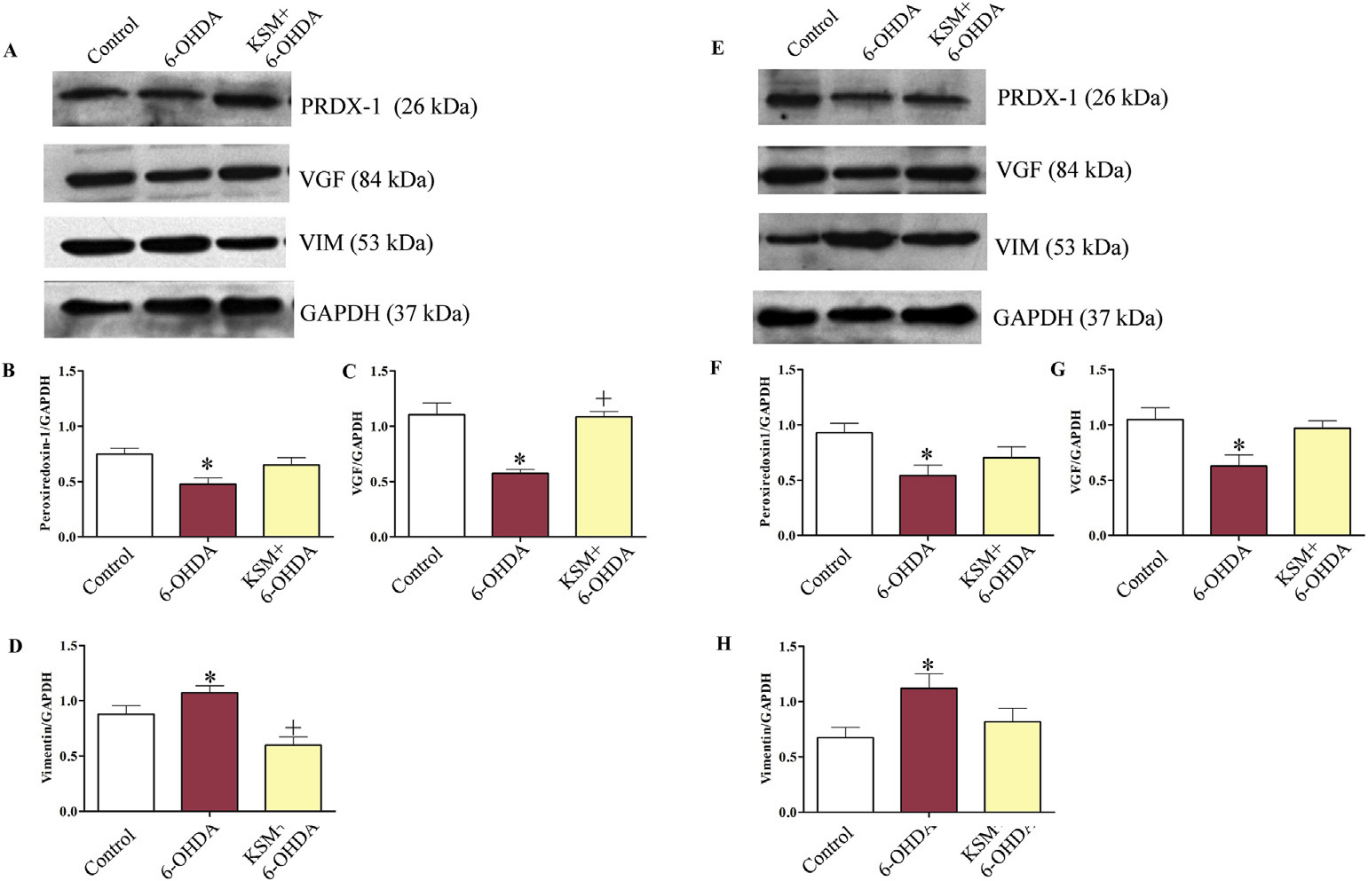
Pre/post-treatment effect of KSM-66 on expression of peroxiredoxin I, vimentin and VGF proteins. In the pre-treatment study, SH-SY5Y cells were pre-treated with 0.5 mg/ml KSM-66 for 24 h followed by incubation with 100 μM 6-OHDA for 2 h. Cellular lysates were prepared, subjected to western blot analysis and analyzed for the presence of oxidative stress proteins (A). Protein band intensities from (A) were quantitated for (B) peroxiredoxin I, (C) VGF and (D) vimentin using ImageJ image analysis software. In the post-treatment study SH-SY5Y cells were incubation with 100 μM 6-OHDA for 2 h then post-treated with 0.5 mg/ml KSM-66 for 24 h. Cellular lysates were prepared, subjected to western blot analysis and analyzed for the presence of oxidative stress proteins (E). Protein band intensities from (E) were quantitated for (F) peroxiredoxin I, (G) VGF and (H) vimentin using ImageJ image analysis software. The expression of all proteins was normalized to GAPDH. Bars represent mean ± SEM of two independent experiments performed in duplicate. Data were analyzed by One-way ANOVA with Bonferroni's multiple comparison test. ∗*p* < 0.05 represents significant differences compared to the control, ^+^*p* < 0.05 represents significant differences compared to 6-OHDA exposed cells. Full blots are shown in supplemental files for 6A as Suppl 6A full blot PRDX-1, Suppl 6A full blot VGF, Suppl 6A full blot VIM and Suppl 6A full blot GAPDH. Full blots are shown in supplemental files for 6E as Suppl 6E full blot PRDX-1, Suppl 6E full blot VGF, Suppl 6E full blot VIM and Suppl 6E full blot GAPDH.

In pre-treatment studies, SH-SY5Y cells were pre-treated with 0.5 mg/ml KSM-66 for 24 h. Then the cells were stimulated with 100 μM 6-OHDA for 2 h compared with control and SH-SY5Y cells that were not pre-treated with KSM-66. The cell extracts were prepared and analyzed for the expression of the three proteins by Western blotting (Figure 6A). The intensity of proteins was normalized by a GAPDH loading control and were analyzed for differences of expression by Prism 5 software. After 6-OHDA stimulation, SH-SY5Y cells, without pre-added with KSM-66, had peroxiredoxin-1 and VGF levels significantly lower than the untreated control (*p* < 0.05) (Figure 6B and C). Interestingly, SH-SY5Y cells pretreated with 0.5 mg/ml KSM-66 possessed similar peroxiredoxin-1 compared to control and significantly higher VGF level compared to the non-pretreatment group (*p* < 0.05) (Figure 6B and C). In addition, 6-OHDA stimulation increased vimentin expression in the non-pretreatment group significantly (*p* < 0.05) (Figure 6D). Pre-treatment with KSM-66 decreased level of vimentin upon 6-OHDA stimulation significantly (*p* < 0.05), compared to the non-pretreatment group (Figure 6D).

In the post-treatment study, SH-SY5Y cells were stimulated with 100 μM 6-OHDA for 2 h and then to rescue the cell damage cells were treated with 0.5 mg/ml KSM-66 for 24 h (Figure 6E). It was found that the non-treatment group had peroxiredoxin-1 and VGF expression significantly lower than the control group (*p* < 0.05) (Figure 6F and G). KSM-66 treatment slightly increased peroxiredoxin-1 compared to the non-treatment group. KSM-66 also restored VGF level similar to control. For vimentin detection, the 6-OHDA group had significantly higher vimentin compared to control and post-treatment with KSM-66 decreased vimentin to the same level as control (Figure 6H).

### 3.5 KSM-66 decreases protein glutathionylation in SH-SY5Y cells

**Figure 7:**
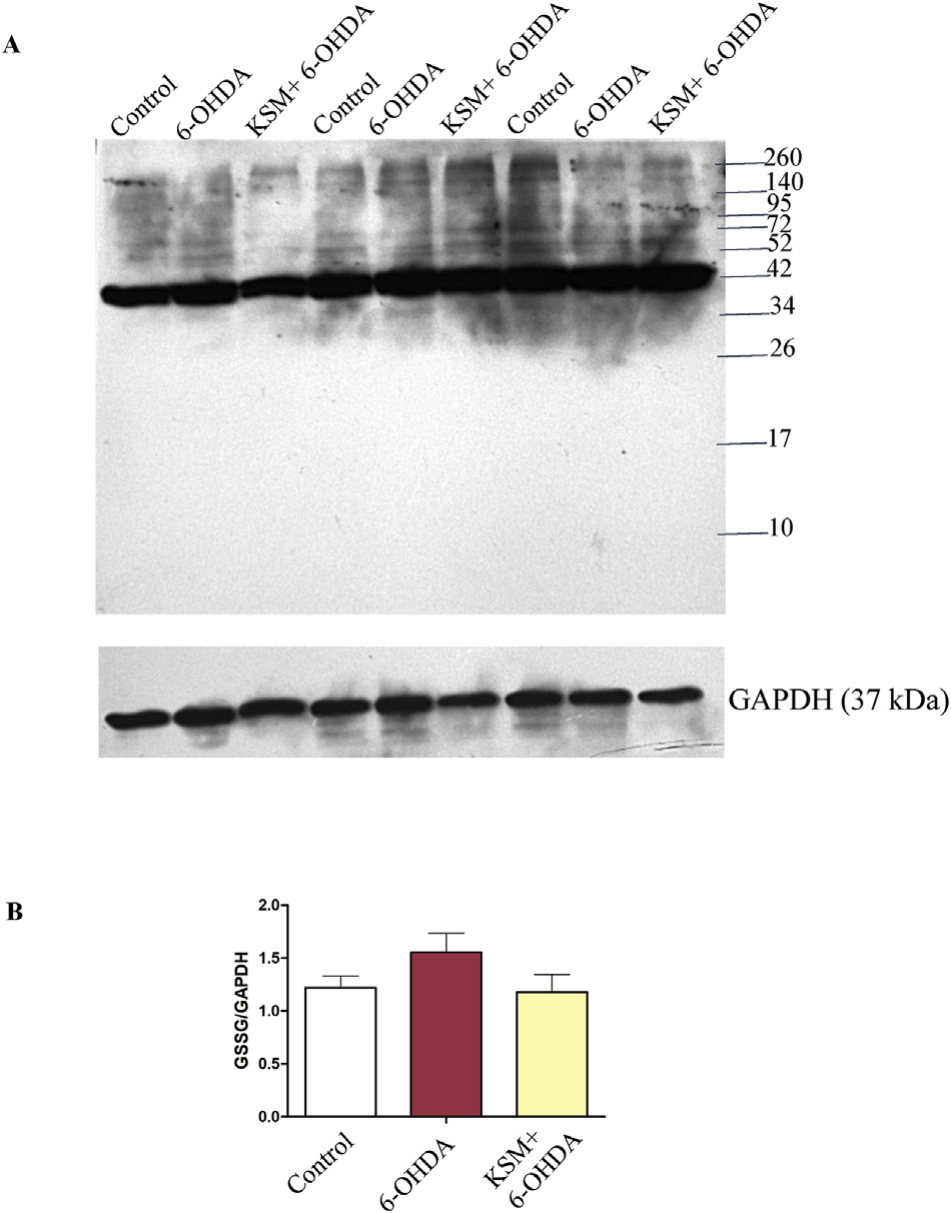
KSM-66 inhibits protein glutathionylation. (A) Glutathionylation levels were detected by using Western blot. Cells were pre-treated with or without 0.5 mg/ml KSM-66 for 24 h followed by 100 μM 6-OHDA for 15 min. Total proteins were separated on 12% SDS-gel under non-reducing conditions (no DTT) for Western blot analysis using anti-glutathione mouse monoclonal antibody. (B) Relative major glutathionylated band (GSSG) was normalized with GAPDH loading. Data are mean ± SEM of three independent experiments. Data were analyzed by One-way ANOVA with Bonferroni's multiple comparison test. No significant differences (*P* > 0.05) in expression profiles were observed for control, 6-OHDA and KSM treatments.

Glutathionylation is the covalent modification of a cysteine residue with glutathione to protect against protein oxidation or modulate protein function. Western blotting was performed to investigated whether KSM-66 would affect the amount of protein glutathionylation in SH-SY5Y cells upon 6-OHDA treatment. As the protein glutathionylation reaction is relatively fast, we therefore treated the cells with 100 μM 6-OHDA for 15 min to compare the oxidation-induced glutathionylation in SH-SY5Y cells that were not pre-treated as well as pre-treated with KSM-66. Figure 7 shows that the control cells had many protein bands that were glutathionylated at baseline conditions. The major glutathionylated band had a size of approximately 45 kDa which can be observed in all cell groups. For intensity comparison, this protein band was chosen for glutathionylation amounts between the three treatment groups. It was found that protein glutathionylation increased upon 6-OHDA treatment. Interestingly, 0.5 mg/ml KSM-66 pretreated SH-SY5Y cells had lower protein glutathionylation than with 6-OHDA treatment alone. The results suggested that KSM-66 can prevent deleterious protein glutathionylation.

### 3.6 KSM-66 attenuates 6-OHDA-induced mitochondrial dysfunction in SH-SY5Y cells

**Figure 8:**
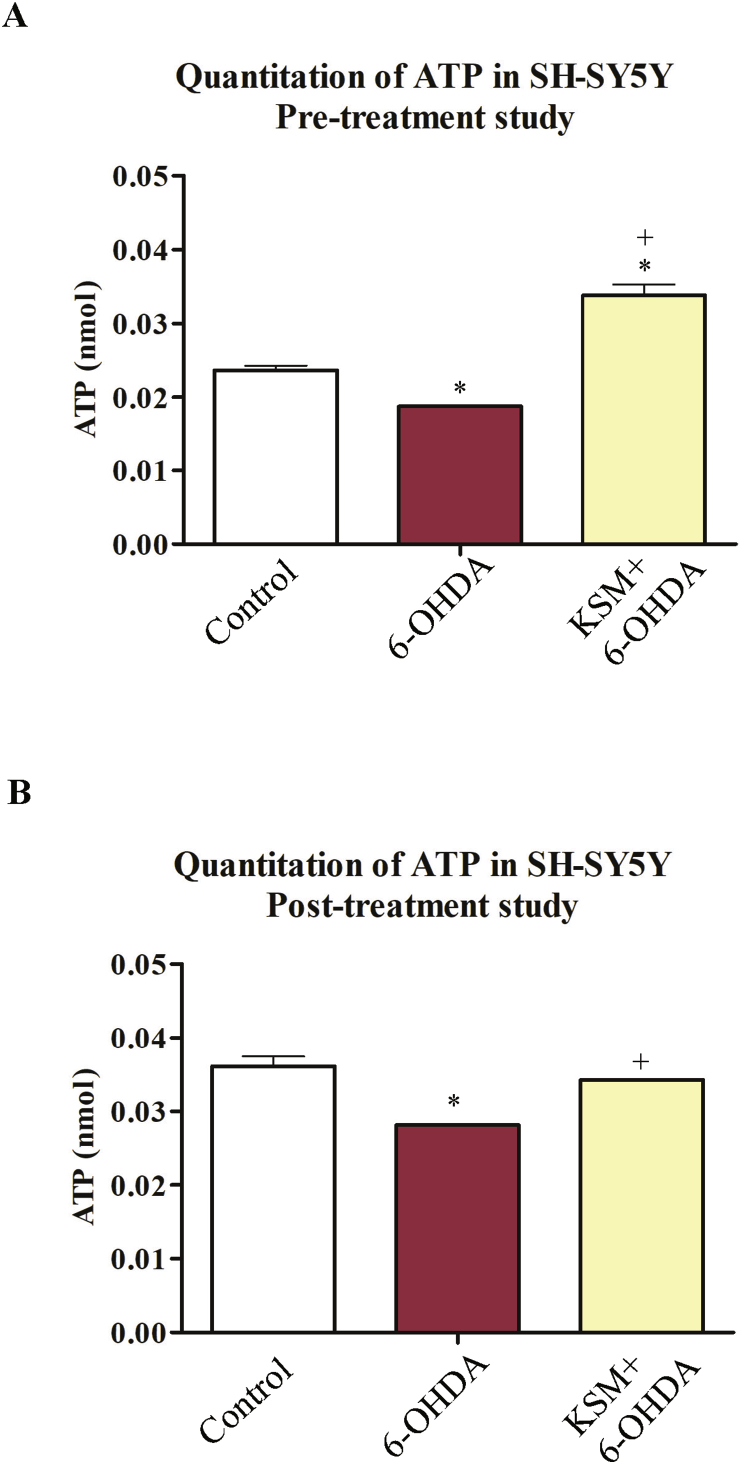
Effect of KSM-66 pre/post-treatment on ATP content during 6-OHDA induced toxicity. In pre-treatment (A) SH-SY5Y cells were treated with 0.5 mg/ml KSM-66 for 24 h then incubated with 100 μM 6-OHDA for 2 h. In post-treatment (B) SH-SY5Y cells were incubated with 100 μM 6-OHDA for 2 h then post-treated with 0.5 mg/ml KSM-66 for 24 h. The ATP content was determined. Values are mean ± SEM of two independent experiments performed in duplicate. Data were analyzed by One-way ANOVA with Bonferroni's Multiple Comparison test; ∗Statistically significant at *P* < 0.05 compared to control. ^+^Statistically significant at *P* < 0.05 compared to 6-OHDA.

To further assess the effects of KSM-66 on mitochondrial injury induced by 6-OHDA, we analyzed intracellular ATP levels in the 6-OHDA-treated cells in both pre- and post-treatment with KSM-66. In the pre-treatment experiment, the ATP amounts in the cells treated with 6-OHDA without KSM-66 pre-added was significantly reduced compared to control cells at 2 h incubation time (Figure 8A). In contrast, KSM-66 pre-treatment remarkably increased intracellular ATP levels upon 6-OHDA treatment. Consistent with that result, KSM-66 post-treatment significantly restored the ATP depletion in the 6-OHDA-treated cells (Figure 8B).

### 3.7 Summary of results

The IC_50_ 24 h values of 6-OHDA and KSM-66 were 145.464 μM and 2.66 mg/ml respectively. The concentration of KSM-66 at 1 mg/ml was chosen for neuroprotection study. In cell viability experiments post-treatment with KSM-66 with 0.25, 0.50, 0.75 and 1 mg/ml revealed that all concentrations significantly increased cell viability compared to 6-OHDA only. Pretreatment with KSM-66 for 24 h resulted in higher cell viability compared to 30-min pretreatment time. All concentrations of KSM-66 significantly increased the cell survival compared to 6-OHDA only treated cells. The cell morphology of pretreatment and post-treatment groups also supported the cell viability results. Pre-treatment with KSM-66 at all concentrations yielded intact cells with higher density than post-treatment conditions. The antioxidant enzyme activity studies showed pre/post treatment with KSM-66 significantly increased peroxidase activity compared to 6-OHDA treated and untreated control cells. The antioxidant protein response showed pre-treatment with KSM-66 significantly increased VGF and decreased vimentin compared to 6-OHDA treated cells. Post-treatment did not show any differences for antioxidant response proteins measured. Pre-treatment with KSM-66 decreased glutathionylated proteins to similar levels as untreated controls. Intracellular ATP amounts were quantitated and showed that pre-treatment increased cellular ATP levels significantly compared to 6-OHDA only and untreated control cells. Post-treatment results demonstrated a similar trend with increasing ATP amounts compared to 6-OHDA only treated cells.

## 4 Discussion

KSM-66, *W. somnifera* extract, commonly known as Ashwagandha, was employed in this study to determine anti-oxidant protection in the Parkinson's model SH-SY5Y cells tested by treatment with the oxidant 6-OHDA. Percent cell viability, anti-oxidant enzyme activities, oxidative stress protein expression, protein glutathionylation detection and ATP levels were studied. We demonstrated that 6-OHDA at 100 μM induced 50% cell survival at 24 h. It also decreased ATP production approximately 20% (Figure 8B) suggesting that 6-OHDA is cytotoxic as well as a mitochondrial inhibitor. Cell viability increased in SH-SY5Y cells treated with 6-OHDA for both post and pre-treatment with KSM-66 (Figure 3C and F) suggesting that KSM-66 had both protective and therapeutic effects in cells exposed to oxidative stress. The results obtained from this study supported previous reports showing the neuroprotective effects of Ashwagandha extract in both *in vitro* and *in vivo* studies (Prakash et al., 2014; Saykally et al., 2017; Vidyashankar et al., 2014). Root extract of Ashwagandha decreased Bisphenol A (BPA) toxicity in HepG2 cells and increased ATP production (Vidyashankar et al., 2014). Alcoholic extract and water extract of Ashwagandha increased human neuroblastoma and rat glioblastoma cell viability exposed to H_2_O_2_ by 20–30%. In addition, upon cell injury by nitrogen gas pulse, root extract from Ashwagandha decreased LDH levels by 20% compared to injured SH-SY5Y controls (Saykally et al., 2017). Apoptotic pathways in dopaminergic neurons of PD mouse model were inhibited by Ashwagandha. Paraquat and maneb, herbicide and fungicide respectively, induce selective damage of dopaminergic neurons. A mouse model of PD co-treated with ethanolic root extract of Ashwagandha and paraquat or maneb was shown to modulate apoptotic signaling with decreased Bax and increased Bcl-2 protein expression (Prakash et al., 2014). Therefore, further research is required to elucidate survival mechanisms when KSM-66 is employed as pre- or post-treatment in neuronal cells.

Although peroxiredoxin I protein expression was not significantly different in 6-OHDA treated cells in both pre/post treatments with KSM-66 (Figure 6). The treatments showed a slight increase in the levels of protein expression. In addition, glutathione peroxidase enzyme activity (Figure 5) supported the results since pre-treatment of the cells with KSM-66 protected the cells from peroxide damage by increasing peroxidase activity in a dose-dependent manner (Figure 5A). Whereas, post-treatment of KSM-66 restored peroxidase activity at 0.5 and 1 mg/ml (Figure 5D) showing that KSM-66 increased peroxidase activity in neuronal cells under oxidative stress. In agreement with previous reports in HepG2 and striatum cells, glutathione peroxidase activities were replenished by Ashwagandha under oxidative stress (Ahmad et al., 2005; Vidyashankar et al., 2014).

Although pre-treatment HED thioltransferase activity was not significantly different to the untreated group, the increased activity was similar to the thioltransferase activity of the control (Figure 5B). The thioltransferase activity of post-treatment also was similar to the control group (Figure 5E) suggesting a possible Omega GST role is to combat oxidative stress in the neuron. Glutathione transferase Omega, previously also called dehydroascorbate reductase, specifically catalyzes thioltransferase reactions including protein glutathionylation (Whitbread et al., 2005). Increased Omega GST *in vivo* expression in neuronal and brain cells have been reported but their role was not elucidated (Fornai et al., 2001; Werner et al., 2008). GST omega was detected in rat substantia nigra by using immunohistochemistry and a putative role described in prevention of DNA oxidative damage and regulation of gene transcription in the brain (Fornai et al., 2001). In addition, GST omega had greater expression in PD than in controls (Werner et al., 2008). Rescue of DmGSTO1A, Drosophila GSTO1A, in park1 mutants which have parkin pathogenic phenotypes, increases the level of glutathionylated ATP synthase β subunit and restores mitochondrial F_1_F_0_-ATP synthase activity. Omega GST also partially rescued the mutant phenotype and the degeneration of DA neurons (Kim et al., 2012). From our previous report, ATP synthase was detected in a glutathionylation blot after SH-SY5Y cells were treated with 6-OHDA (Wongtrakul et al., 2018). The protein showed significant decreased glutathionylation compared to untreated cells. The cells transfected with hGSTO1-1 had only decreased glutathionylated vimentin suggesting that ATP synthase was expressed to the same extent as in untreated cells. The present study demonstrated increased intra-cellular ATP after treatment with KSM66 (Figure 8) suggesting that KSM66 extract enhanced and replenished omega GST enzyme to combat oxidative stress which resulted in increased thioltransferase activity and ATP content.

Our experiments (Figure 6) demonstrate increased expression levels of vimentin after 6-OHDA treatment. Pre-treatment of KSM-66 significantly decreased vimentin in the presence of 6-OHDA treatment (Figure 6D) in addition, post-treatment of KSM-66 after 6-OHDA treatment had decreased vimentin to a similar amount as control cells (Figure 6H). Our results support the role of withanolide in anti-inflammatory effect (Wei et al., 2019). Withanferin A, a withanolide substance, inhibits NF-κB activation and targets vimentin protein. A previous study reported that 6-OHDA decreased VGF in SH-SY5Y neuronal cells (Cocco et al., 2020). VGF antibody employed in this study is specific to c-t peptide as previously reported. Our study found that pre-treatment of the cells with KSM-66 significantly increased VGF compared to 6-OHDA stimulated cells (Figure 6C). KSM-66 also restored VGF levels in the post-treatment study although the level was not significantly different from 6-OHDA stimulated cells (Figure 6G). The role of KSM in VGF stimulation therefore requires further study.

6-OHDA treated cells showed a slight increase in protein glutathionylation (Figure 7). The main cause of glutathionylation gain during oxidative stress in SH-SY5Y is due to the formation of protein glutathione mixed disulphides. Increased glutathionylation levels are an indicator of high oxidative stress and protein inactivation and damage (Ghezzi, 2013). Interestingly, pre-treatment of SH-SY5Y cells with KSM-66 followed by 6-OHDA treatment was able to lower protein glutathionylation within the cells (Figure 7). These results are in agreement with earlier reports on Ashwagandha extract mediated inhibition of glutathionylation formation in *in vitro* and *in vivo* studies (Sood et al., 2018; Vidyashankar et al., 2014). It has been reported that a mechanism for Ashwagandha protection of neuronal cells was through activation of the Nrf2 pathway (Hybertson et al., 2019; Priyandoko et al., 2011). The studies showed that Ashwagandha protects cells from oxidative stress by the Nrf2 pathway upregulation of cytoprotective genes (Hybertson et al., 2019) and Keap-Nrf2-ARE signaling (Priyandoko et al., 2011). Ashwagandha appears to cause increased Nrf2 expression and translocation to the nucleus. Nrf2's binding on ARE causes the induction of several phase I and phase II metabolizing enzymes, phase III detoxifying proteins and antioxidant proteins. Upregulation of detoxification enzymes enhances cell survival and protection due to an improved redox state which prevents glutathionylated protein accumulation in SH-SY5Y cells. Additional evidence of Ashwagandha mediated neuroprotection was reported showing that withanolide A increased glutathione synthesis in neuronal cells by upregulating GCLC levels through the Nrf2 pathway in a corticosterone dependent manner during hypoxia (Baitharu et al., 2014).

In conclusion KSM-66, *W. somnifera* root extract, demonstrated neuroprotective effects showing a capacity to increase the viability of SH-SY5Y cells, a Parkinson's Disease model, to increase glutathione peroxidase and thiol transferase enzyme activities, to modulate the expression of oxidative stress response proteins; peroxiredoxin I, VGF and vimentin, to increase intracellular ATP levels and to modulate redox regulation by decreasing glutathionylated protein levels. These results suggest that KSM-66 is a promising lead drug target for modulating cell damage and cell death due to oxidative stress. Further studies to elucidate the KSM-66 active components and the mechanisms of action are required.

## Declarations

### Author contribution statement

Jeerang Wongtrakul: Conceived and designed the experiments; Performed the experiments; Analyzed and interpreted the data; Contributed reagents, materials, analysis tools or data; Wrote the paper.

Thananya Thongtan: Conceived and designed the experiments; Performed the experiments; Analyzed and interpreted the data; Contributed reagents, materials, analysis tools or data.

Benjawan Kumrapich: Performed the experiments.

Chonticha Saisawang: Contributed reagents, materials, analysis tools or data.

Albert J. Ketterman: Conceived and designed the experiments; Analyzed and interpreted the data; Wrote the paper.

### Funding statement

This study was supported by BRAND’S Health Research Foundation for Thai Society and Brand Suntory (Thailand) Co., Ltd.

### Data availability statement

Data included in article/supplementary material/referenced in article.

### Declaration of interests statement

The authors declare no conflict of interest.

### Additional information

No additional information is available for this paper.

## Figures and Tables

**Suppl 6A full blot GAPDH:**
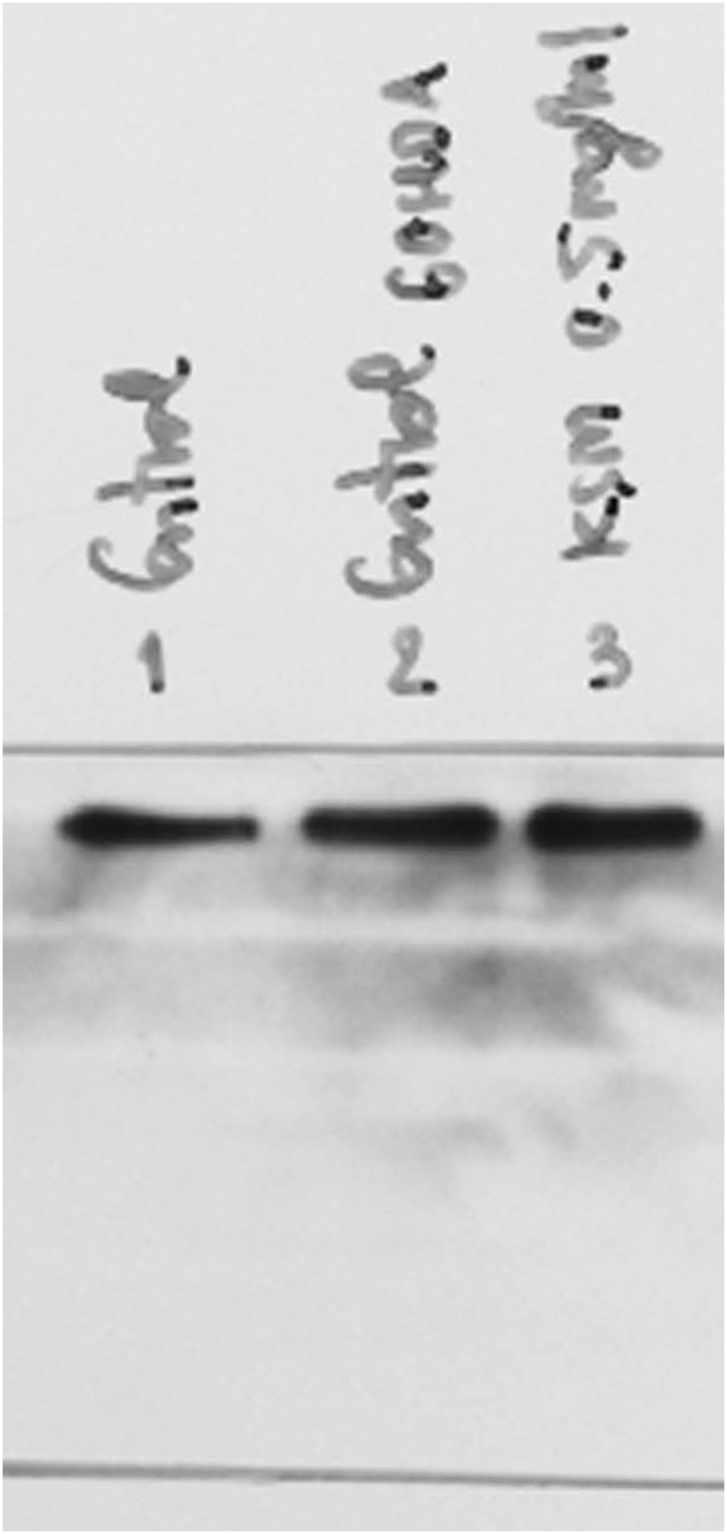


**Suppl 6A full blot PRDX-1:**
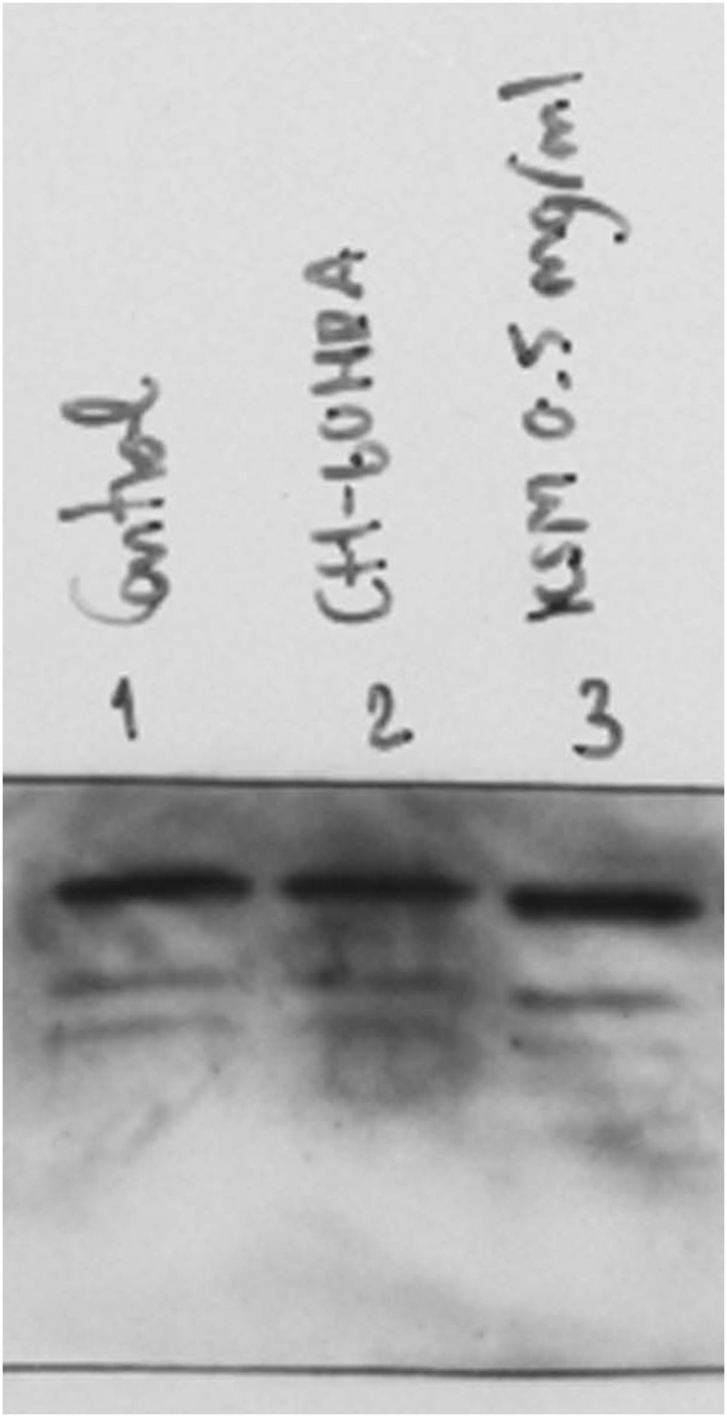


**Suppl 6A full blot VGF:**
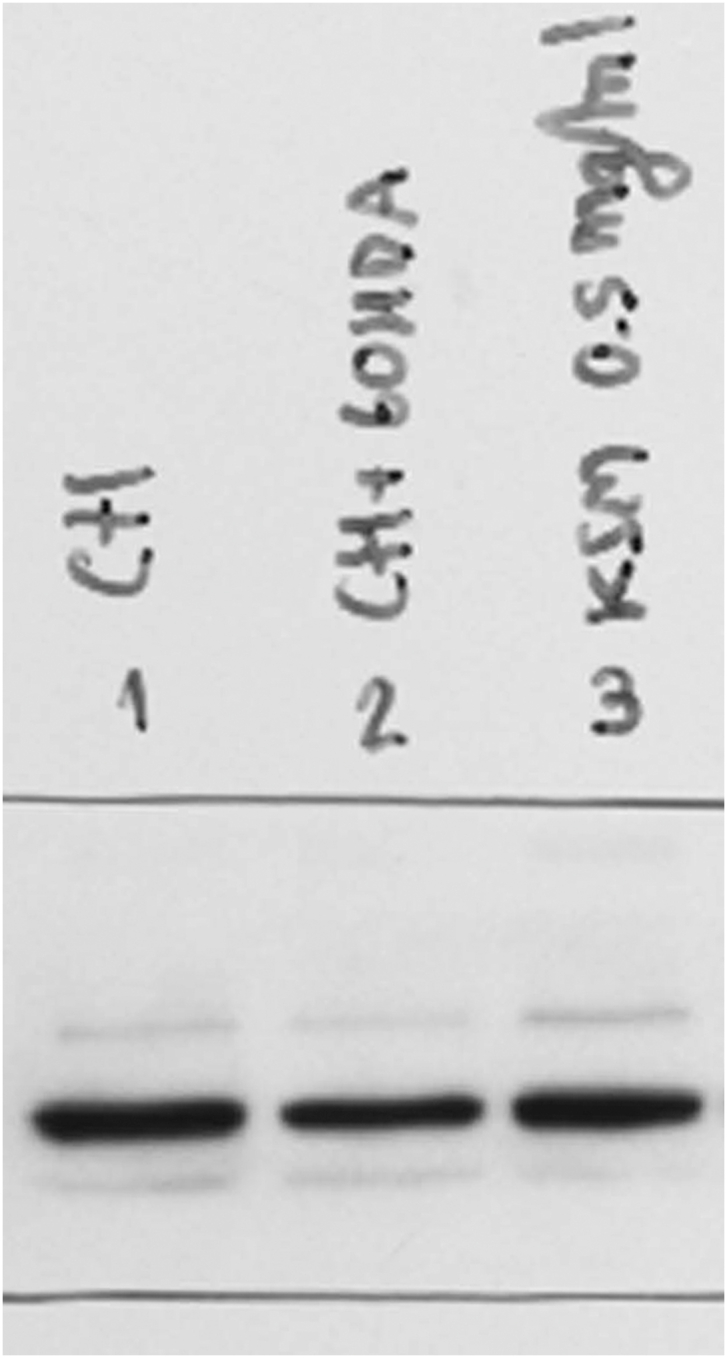


**Suppl 6A full blot VIM:**
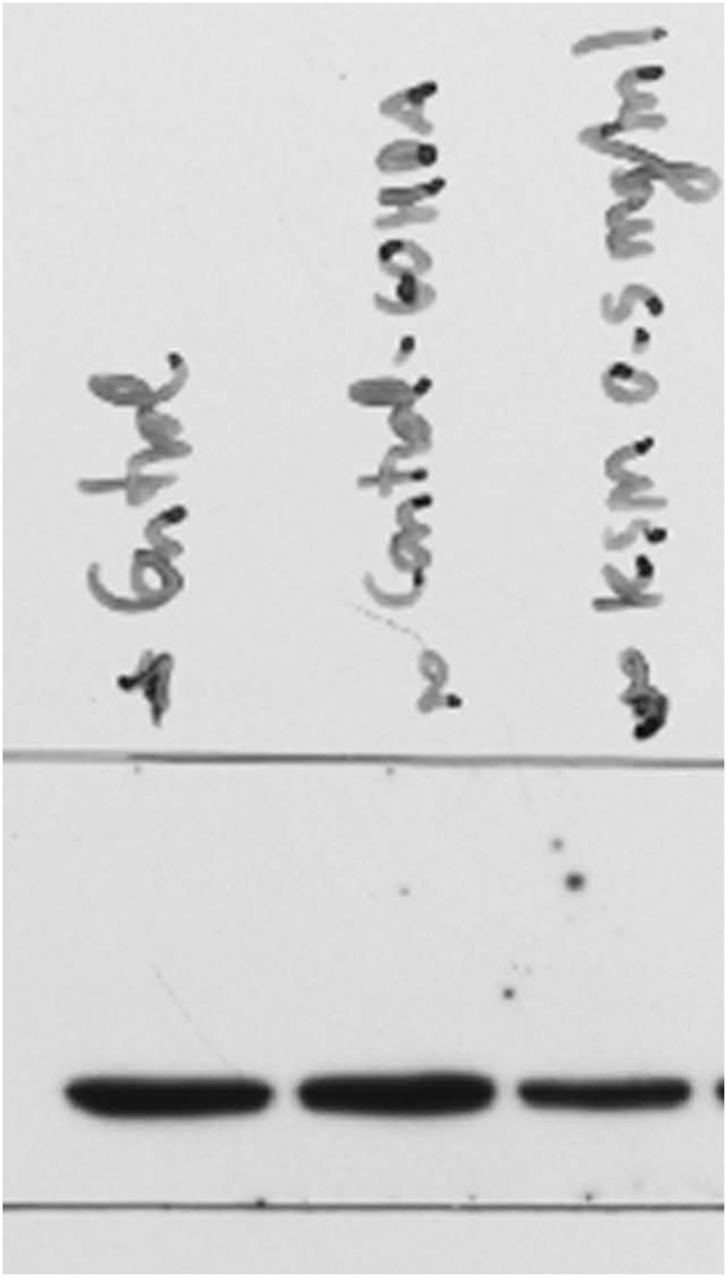


**Suppl 6E full blot GAPDH:**
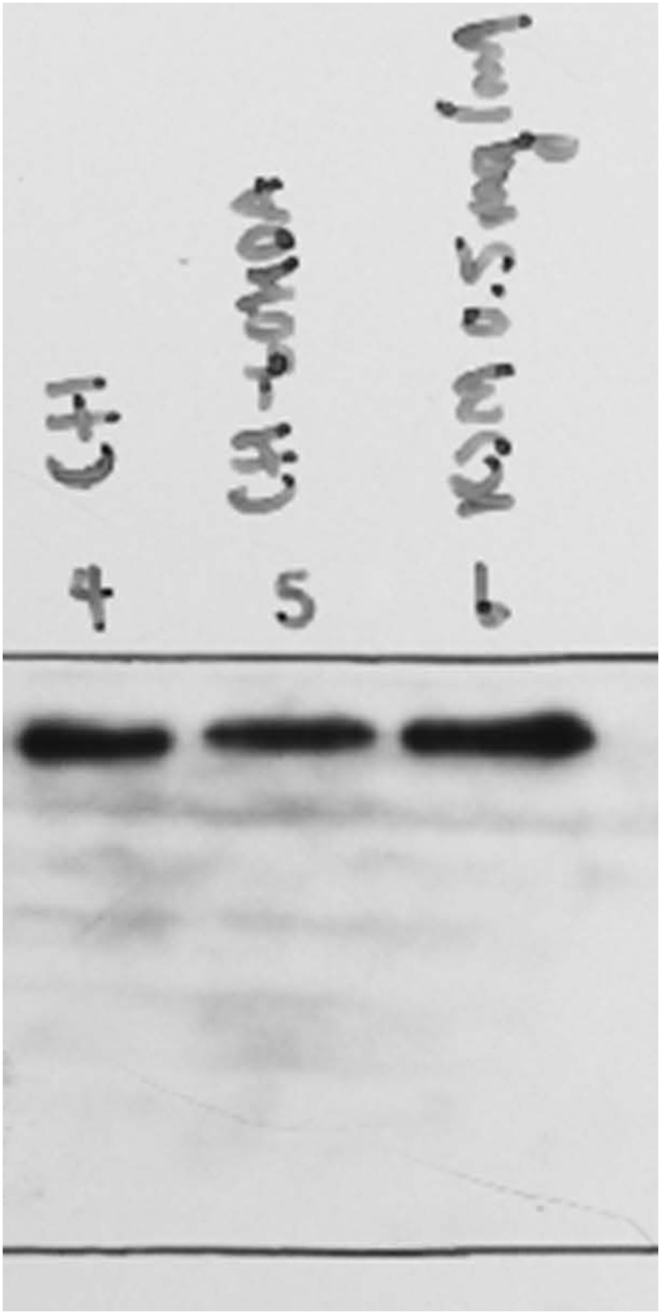


**Suppl 6E full blot PRDX-1:**
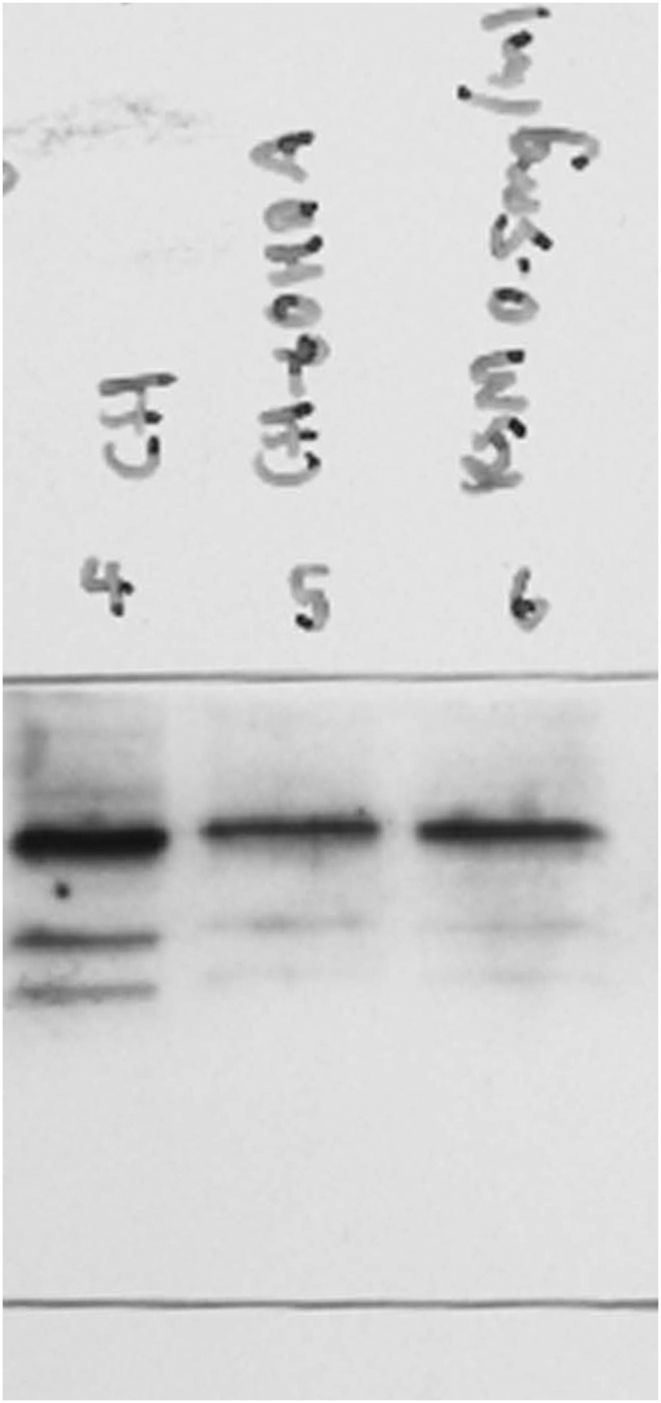


**Suppl 6E full blot VGF:**
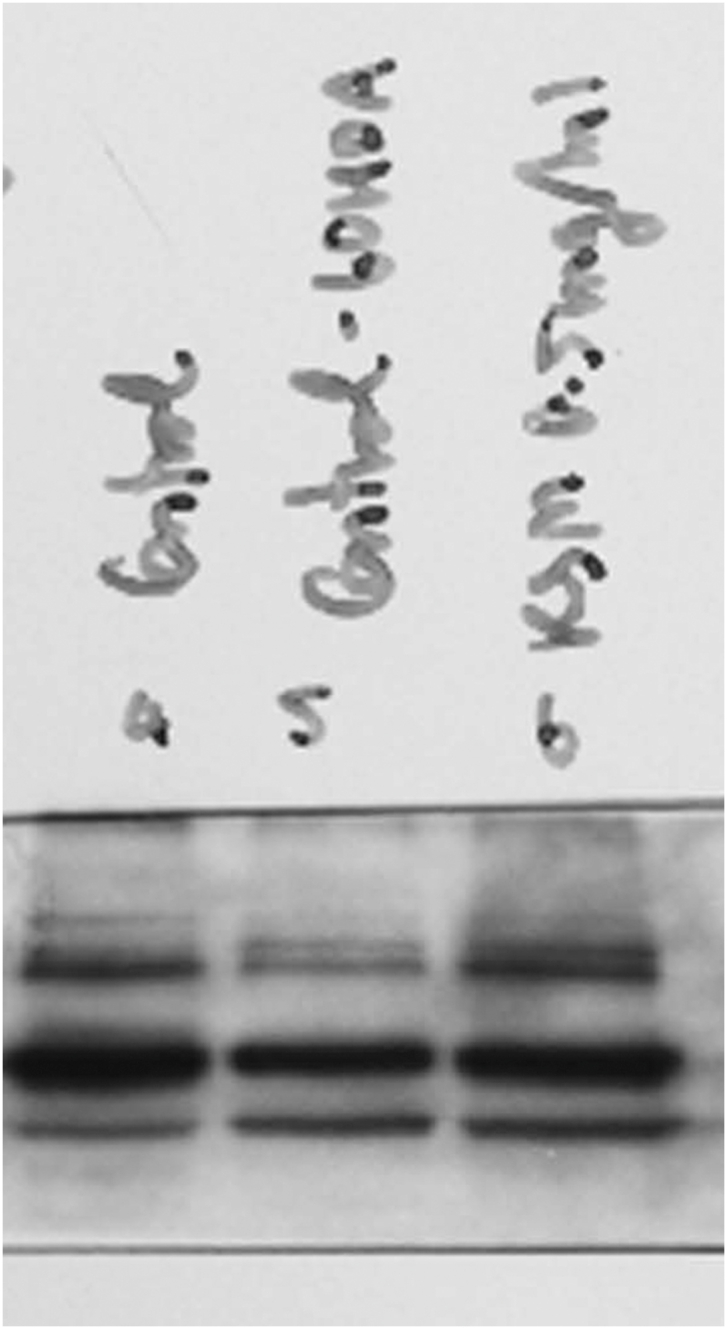


**Suppl 6E full blot VIM:**
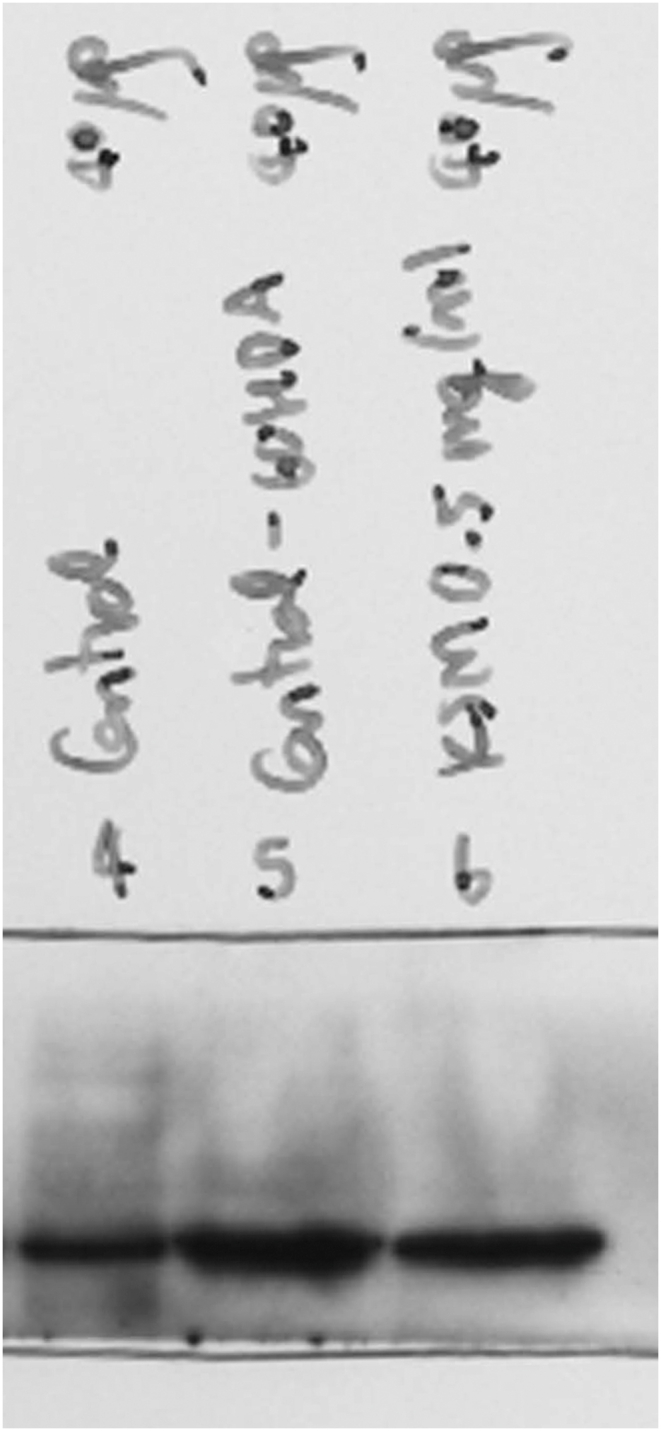


## References

[bib1] Ahmad M., Saleem S., Ahmad A.S., Ansari M.A., Yousuf S., Hoda M.N., Islam F. (2005). Neuroprotective effects of Withania somnifera on 6-hydroxydopamine induced Parkinsonism in rats. Hum. Exp. Toxicol..

[bib2] Ahmed W., Mofed D., Zekri A.R., El-Sayed N., Rahouma M., Sabet S. (2018). Antioxidant activity and apoptotic induction as mechanisms of action of Withania somnifera (Ashwagandha) against a hepatocellular carcinoma cell line. J. Int. Med. Res..

[bib3] Akhoon B.A., Pandey S., Tiwari S., Pandey R. (2016). Withanolide A offers neuroprotection, ameliorates stress resistance and prolongs the life expectancy of Caenorhabditis elegans. Exp. Gerontol..

[bib4] Baitharu I., Jain V., Deep S.N., Shroff S., Sahu J.K., Naik P.K., Ilavazhagan G. (2014). Withanolide A prevents neurodegeneration by modulating hippocampal glutathione biosynthesis during hypoxia. PLoS One.

[bib5] Bhasin S., Singh M., Singh D. (2019). Review on bioactive metabolites of Withania somnifera. (L.) Dunal and its pharmacological significance. J. Pharmacogn. Phytochem..

[bib6] Blandini F., Armentero M.T., Martignoni E. (2008). The 6-hydroxydopamine model: news from the past. Park. Relat. Disord..

[bib7] Cocco C., Corda G., Lisci C., Noli B., Carta M., Brancia C., Manca E., Masala C., Marrosu F., Solla P. (2020). VGF peptides as novel biomarkers in Parkinson's disease. Cell Tissue Res..

[bib8] Cocco C., D'Amato F., Noli B., Ledda A., Brancia C., Bongioanni P., Ferri G.L. (2010). Distribution of VGF peptides in the human cortex and their selective changes in Parkinson's and Alzheimer's diseases. J. Anat..

[bib9] Collaborators G.P.s.D. (2018). Global, regional, and national burden of Parkinson's disease, 1990-2016: a systematic analysis for the Global Burden of Disease Study 2016. Lancet Neurol..

[bib10] Collinson E.J., Grant C.M. (2003). Role of yeast glutaredoxins as glutathione S-transferases. J. Biol. Chem..

[bib11] Danielsson F., Peterson M.K., Caldeira Araujo H., Lautenschlager F., Gad A.K.B. (2018). Vimentin diversity in health and disease. Cells.

[bib12] Dar N.J., Hamid A., Ahmad M. (2015). Pharmacologic overview of Withania somnifera, the Indian ginseng. Cell. Mol. Life Sci..

[bib13] Dorsey E.R., Sherer T., Okun M.S., Bloem B.R. (2018). The emerging evidence of the Parkinson pandemic. J. Parkinsons Dis..

[bib14] Ferri G.L., Noli B., Brancia C., D'Amato F., Cocco C. (2011). VGF: an inducible gene product, precursor of a diverse array of neuro-endocrine peptides and tissue-specific disease biomarkers. J. Chem. Neuroanat..

[bib15] Fornai F., Gesi M., Saviozzi M., Lenzi P., Piaggi S., Ferrucci M., Casini A. (2001). Immunohistochemical evidence and ultrastructural compartmentalization of a new antioxidant enzyme in the rat substantia nigra. J. Neurocytol..

[bib16] Ghezzi P. (2013). Protein glutathionylation in health and disease. Biochim. Biophys. Acta.

[bib17] Habig W.H., Pabst M.J., Jakoby W.B. (1974). Glutathione S-transferases. The first enzymatic step in mercapturic acid formation. J. Biol. Chem..

[bib18] Henderson-Smith A., Corneveaux J.J., De Both M., Cuyugan L., Liang W.S., Huentelman M., Adler C., Driver-Dunckley E., Beach T.G., Dunckley T.L. (2016). Next-generation profiling to identify the molecular etiology of Parkinson dementia. Neurol. Genet..

[bib19] Hernandez-Baltazar D., Zavala-Flores L.M., Villanueva-Olivo A. (2017). The 6-hydroxydopamine model and parkinsonian pathophysiology: novel findings in an older model. Neurologia.

[bib20] Hiratsuka A., Yamane H., Yamazaki S., Ozawa N., Watabe T. (1997). Subunit Ya-specific glutathione peroxidase activity toward cholesterol 7-hydroperoxides of glutathione S-transferases in cytosols from rat liver and skin. J. Biol. Chem..

[bib21] Hybertson B.M., Gao B., Bose S., McCord J.M. (2019). Phytochemical combination PB125 activates the Nrf2 pathway and induces cellular protection against oxidative injury. Antioxidants.

[bib22] Kaur N., Niazi J., Bains R. (2013). A review on pharmacological profile of Withania somnifera (ashwagandha). Res. Rev. J. Bot. Sci..

[bib23] Kim K., Kim S.H., Kim J., Kim H., Yim J. (2012). Glutathione s-transferase omega 1 activity is sufficient to suppress neurodegeneration in a Drosophila model of Parkinson disease. J. Biol. Chem..

[bib24] Kuboyama T., Tohda C., Komatsu K. (2005). Neuritic regeneration and synaptic reconstruction induced by withanolide A. Br. J. Pharmacol..

[bib25] Levin E.C., Acharya N.K., Sedeyn J.C., Venkataraman V., D'Andrea M.R., Wang H.Y., Nagele R.G. (2009). Neuronal expression of vimentin in the Alzheimer's disease brain may be part of a generalized dendritic damage-response mechanism. Brain Res..

[bib26] Maserejian N., Vinikoor-Imler L., Dilley A. (2020). Estimation of the 2020 global population of Parkinson’s disease (PD) [abstract]. Mov. Disord..

[bib27] Mishra B., Sangwan R.S., Mishra S., Jadaun J.S., Sabir F., Sangwan N.S. (2014). Effect of cadmium stress on inductive enzymatic and nonenzymatic responses of ROS and sugar metabolism in multiple shoot cultures of Ashwagandha (Withania somnifera Dunal). Protoplasma.

[bib28] Mishra L.C., Singh B.B., Dagenais S. (2000). Scientific basis for the therapeutic use of Withania somnifera (ashwagandha): a review. Alternative Med. Rev..

[bib29] Monico A., Duarte S., Pajares M.A., Perez-Sala D. (2019). Vimentin disruption by lipoxidation and electrophiles: role of the cysteine residue and filament dynamics. Redox Biol..

[bib30] Morales I., Sanchez A., Rodriguez-Sabate C., Rodriguez M. (2016). The astrocytic response to the dopaminergic denervation of the striatum. J. Neurochem..

[bib31] Prakash J., Chouhan S., Yadav S.K., Westfall S., Rai S.N., Singh S.P. (2014). Withania somnifera alleviates parkinsonian phenotypes by inhibiting apoptotic pathways in dopaminergic neurons. Neurochem. Res..

[bib32] Priyandoko D., Ishii T., Kaul S.C., Wadhwa R. (2011). Ashwagandha leaf derived withanone protects normal human cells against the toxicity of methoxyacetic acid, a major industrial metabolite. PLoS One.

[bib33] Rai S.N., Birla H., Walia Z.,., Saumitra S.S., Singh S.P. (2018). The role of mucuna pruriens and Withania somnifera in the neuroprotection and treatment of Parkinson’s disease. SOJ Neurol..

[bib34] Rayees S., Malik F., Kaul S.C., Wadhwa R. (2017). Science of Ashwagandha: Preventive and Therapeutic Potentials.

[bib35] Rhee S.G. (2016). Overview on peroxiredoxin. Mol. Cell..

[bib36] Rhee S.G., Kang S.W., Chang T.S., Jeong W., Kim K. (2001). Peroxiredoxin, a novel family of peroxidases. IUBMB Life.

[bib37] Sabina E.P., Rasool M., Vedi M., Navaneethan D., Ravichander M., Sarah P., Parthasarathy R.T. (2013). Hepatoprotective and antioxidant potential of Withania somnifera against Paracetamol-induced liver damage in rats. Int. J. Pharm. Pharmaceut. Sci..

[bib38] Saykally J.N., Hatic H., Keeley K.L., Jain S.C., Ravindranath V., Citron B.A. (2017). Withania somnifera extract protects model neurons from in vitro traumatic injury. Cell Transplant..

[bib39] Simola N., Morelli M., Carta A.R. (2007). The 6-hydroxydopamine model of Parkinson's disease. Neurotox. Res..

[bib40] Sood A., Mehrotra A., Dhawan D.K., Sandhir R. (2018). Indian Ginseng (Withania somnifera) supplementation ameliorates oxidative stress and mitochondrial dysfunctions in experimental model of stroke. Metab. Brain Dis..

[bib41] van den Pol A.N., Bina K., Decavel C., Ghosh P. (1994). VGF expression in the brain. J. Comp. Neurol..

[bib42] Vegh C., Wear D., Okaj I., Huggard R., Culmone L., Eren S., Cohen J., Rishi A.K., Pandey S. (2021). Combined ubisol-Q10 and ashwagandha root extract target multiple biochemical mechanisms and reduces neurodegeneration in a paraquat-induced rat model of Parkinson's disease. Antioxidants.

[bib43] Vidyashankar S., Thiyagarajan O.S., Varma R.S., Kumar L.M.S., Babu U.V., Patki P.S. (2014). Ashwagandha (Withania somnifera) supercritical CO2 extract derived withanolides mitigates Bisphenol A induced mitochondrial toxicity in HepG2 cells. Toxicol. Rep..

[bib44] Wei Z., Li T., Su H., Wang Q., Kuang H. (2019). Pharmacological effects of withanolides. Open Acc. J. Comp. Alt. Med..

[bib45] Werner C.J., Heyny-von Haussen R., Mall G., Wolf S. (2008). Proteome analysis of human substantia nigra in Parkinson's disease. Proteome Sci..

[bib46] Whitbread A.K., Masoumi A., Tetlow N., Schmuck E., Coggan M., Board P.G. (2005). Characterization of the omega class of glutathione transferases. Methods Enzymol..

[bib47] Wongtrakul J., Saisawang C., Kumrapich B., Wipasa J., Roytrakul S., Ketterman A.J. (2018). Proteomic analysis of human glutathione transferase omega (hGSTO1) stable transfection in a 6-hydroxydopamine-induced neuronal cells. Gen. Physiol. Biophys..

